# Combinatorial batching of DNA for ultralow-cost detection of pathogenic variants

**DOI:** 10.1186/s13073-023-01167-6

**Published:** 2023-03-14

**Authors:** Ulrik Kristoffer Stoltze, Christian Munch Hagen, Thomas van Overeem Hansen, Anna Byrjalsen, Anne-Marie Gerdes, Victor Yakimov, Simon Rasmussen, Marie Bækvad-Hansen, David Michael Hougaard, Kjeld Schmiegelow, Henrik Hjalgrim, Karin Wadt, Jonas Bybjerg-Grauholm

**Affiliations:** 1 Department of Pediatrics and Adolescent Medicine, Rigshospitalet, Blegdamsvej 9, 2100 KBH Ø, Denmark; 2 Department of Clinical Genetics, Rigshospitalet, Blegdamsvej 9, 2100 KBH Ø, Denmark; 3 Department of Congenital Disorders, Statens Serum Institute, 2300 KBH S, Artillerivej 5 Denmark; 4 Department of Clinical Medicine, Copenhagen University, Blegdamsvej 3B, 2200 KBH N, Denmark; 5 Novo Nordisk Foundation Center for Protein Research, Copenhagen University, Blegdamsvej 3B, 2200 KBH N, Denmark; 6 Danish Cancer Society Research Centre, Danish Cancer Society, Strandboulevarden 49, 2100 KBH Ø, Denmark; 7 Department of Epidemiology Research, Statens Serum Institut, 2300 KBH S, Artillerivej 5 Denmark; 8 Department of Haematology, Rigshospitalet, Blegdamsvej 9, 2100 Copenhagen Ø, Denmark

**Keywords:** Germline, Genomics, Population, Neonatal, Screening, Frugal science, Pediatrics, Cancer predisposition, Rare disease, Health care economics

## Abstract

**Background:**

Next-generation sequencing (NGS) based population screening holds great promise for disease prevention and earlier diagnosis, but the costs associated with screening millions of humans remain prohibitive. New methods for population genetic testing that lower the costs of NGS without compromising diagnostic power are needed.

**Methods:**

We developed double batched sequencing where DNA samples are batch-sequenced twice — directly pinpointing individuals with rare variants. We sequenced batches of at-birth blood spot DNA using a commercial 113-gene panel in an explorative (*n* = 100) and a validation (*n* = 100) cohort of children who went on to develop pediatric cancers. All results were benchmarked against individual whole genome sequencing data.

**Results:**

We demonstrated fully replicable detection of cancer-causing germline variants, with positive and negative predictive values of 100% (95% CI, 0.91–1.00 and 95% CI, 0.98–1.00, respectively). Pathogenic and clinically actionable variants were detected in *RB1*, *TP53*, *BRCA2*, *APC*, and 19 other genes. Analyses of larger batches indicated that our approach is highly scalable, yielding more than 95% cost reduction or less than 3 cents per gene screened for rare disease-causing mutations. We also show that double batched sequencing could cost-effectively prevent childhood cancer deaths through broad genomic testing.

**Conclusions:**

Our ultracheap genetic diagnostic method, which uses existing sequencing hardware and standard newborn blood spots, should readily open up opportunities for population-wide risk stratification using genetic screening across many fields of clinical genetics and genomics.

**Supplementary Information:**

The online version contains supplementary material available at 10.1186/s13073-023-01167-6.

## Background

In high-income nations, rare disease (RD) is arguably the leading cause of childhood mortality, responsible for one in three deaths [1]. More than 6000 different types of RD have been identified, and it is estimated that as many as one in 16 individuals under the age of 25 years suffers from some form of RD [2]. This corresponds to a quarter of a billion people globally.

Genetic etiologies have been established for more than 70% of RDs [2], rendering them obvious candidates for genetic screening. In most cases, next-generation DNA sequencing (NGS) technologies can readily detect the pathogenic germline variants that cause RDs, but currently, RD screening is generally limited to the small subset of conditions with established biochemical signatures that can be implied by mass spectroscopy or other techniques [3, 4]. Direct diagnostic genetic testing is reserved for the second tier, but moving the genetic sequencing to the first tier, so-called *genotype-first* screening, would open up opportunities to simultaneously screen for hundreds of rare genetic conditions [5].

However, various technical challenges notwithstanding [6, 7] the mere costs of NGS have effectively prevented its implementation into large-scale screening [8]. Individual sequencing, whether undertaken as single gene, gene-panel, or whole exome/genome sequencing (WES/WGS), would be costly for any population-based initiative. Batching of DNA samples has been suggested as a means to reduce NGS costs; however, low reliability has led to this approach being mostly abandoned [9].

We therefore tested a novel approach to mass genetic screening aimed at overcoming these challenges. We hypothesized that by double batching DNA from all individuals in a population, we could dramatically lower sequencing costs while maintaining high reliability and, most importantly, be able to immediately pinpoint identified rare variants to specific individuals. Approaches similar to double batched sequencing (DoBSeq) have been described and investigated previously [9, 10], but these have been without benchmarking and parameter validation; hence its performance for real-world applications remains wholly unexplored.

We selected participants from a prospective, nation-wide genomic study of childhood cancer [11]. This disease area is but one of many that stands to benefit from NGS-based neonatal screening, yet here we focus only on the screening of cancer predisposition genetics, which may be considered a prototype disease for our method’s broader potential. Highly penetrant cancer predisposition syndromes (CPS) caused by pathogenic germline variants are common in childhood cancer patients [11–13] and are often clinically silent or discrete before cancer symptoms arise, likely contributing to widespread underdiagnosis [14, 15]. Furthermore, pediatric CPS variants are frequently *de novo*, and consequently, the only option for pre-symptomatic diagnosis is screening.

## Methods

Below the methodological details of this study are described briefly. For the purposes of full replicability, please see the extended methods in the supplemental materials.

### Primary endpoint

The primary endpoint of this work was to establish a bioinformatic methodology, which could reliably call cohort-unique, clinically relevant single nucleotide variants (SNVs) and insertions/deletions of fewer than 50 base pairs (indels) in individuals based solely on DobSeq data. This performance was benchmarked against individual whole genome sequencing (WGS).

### Secondary endpoints

Evaluating DoBSeq’s scalability by testing detection rates of cohort-unique, clinically relevant variants in stand-alone batches with an increased number of patients and correspondingly lowered per-allele coverage. Evaluating DoBSeq’s ability to detect any cohort-unique variant regardless of ontology and pathogenicity.

### Cohorts

Both cohorts investigated in this work were a subset of participants from a Danish childhood cancer genomics study called STAGING (Sequencing Tumor and Germline DNA - Implication and National Guidelines) [11]. STAGING offered inclusion to any Danish person younger than 18 years of age at time of cancer diagnosis (all types) since Jan 1^st^ 2017 (on-going study; 439 and 549 participants included at the times of construction of the explorative and validation cohorts, respectively). Recruiting happened at all four pediatric cancer centers in Denmark, where the treating physician, assisted by a project nurse, informed and consented patients, following genetic counseling. As part of the STAGING study, WGS was performed on leukocytic germline DNA as described below. STAGING excluded patients that did not speak Danish or English sufficiently to give informed consent in these languages. Based on this national pediatric cancer cohort, we constructed an explorative cohort of 100 patients, selected, firstly, for the availability of neonatal Guthrie cards (born in Denmark), and, secondly, for the presence of pathogenic germline variants in genes covered by the gene panel described below. Subsequently, we constructed a validation cohort of 100 new patients based on the same national cohort, which were selected for non-inclusion in the explorative cohort, but otherwise identically.

### Gold standard: germline whole-genome sequencing (WGS)

All 200 participants in the explorative and validation cohorts had WGS data from germline DNA available at the outset of the study. Sequencing protocols have been published in detail elsewhere [11]. The WGS variant call files were subsetted to genomic areas corresponding to the gene panel (Additional file 1: Table S1) using bcftools/1.10. This subset constituted the raw gold standard reference for SNV and indel germline variants present in the cohorts (Additional file 1: Fig. S1).

### Double batched sequencing (DobSeq) of neonatal bloodspots

**Fig. 1:**
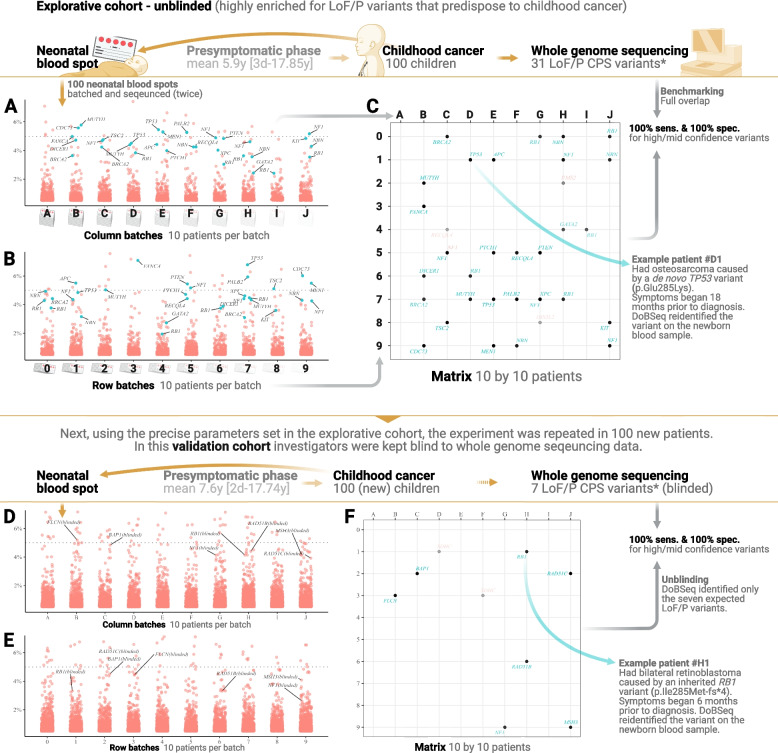
The main findings and performance of double-batched sequencing (DoBSeq) in the explorative and validation cohorts. Upper panel: At the top; a timeline showing how all patients in the explorative cohort had neonatal blood spots taken at birth, followed by a presymptomatic phase prior to a cancer diagnosis (equivalent to neonatal blood sample age), after which they underwent whole genome sequencing which identified several loss-of-function or reported pathogenic variants (LoF/P). **A** Jitter plot showing LoF/P variants detected in the 10-column batches, plotted with variant allele frequency (VAF) on the *y*-axis. Blue and labeled dots represent true positive variants while red and unlabeled dots represent false positives. The gray dotted line represents the theoretically expected VAF of 5% for non-mosaic heterozygous variants (1 of 20 alleles). **B** Jitter plot shows the same as **A** only for row batches. **C** Doubly detected LoF/P variants are pinned to a specific patient in a matrix where each intersection represents one sample/patient. Dots represent variants identified by DoBSeq. Teal gene names indicate true positives (also found on WGS) and red gene names show false positives (not found on WGS). Higher transparency of dots/gene names indicates lower confidence. Lower panel: **D**–**F** are identical to **A**–**C**, only showing data and results from the validation cohort (VC). * The number of LoF/P variants found on whole genome sequencing refers to SNVs and indels within the exonic regions covered by the panel used in the DoBSeq matrices. See the “Methods” section for further details

From each of the 200 participants’ at-birth Guthrie cards, two 3.2-mm discs were stamped out and DNA was extracted on a per-individual basis. For each individual sample, DNA concentrations were measured and then normalized to ensure equimolar contributions of DNA from each individual. Next, the 100 samples from the explorative cohort were randomly allocated with a number from #00 to #99. Volumes equal to 10ng of genomic DNA were taken from each of the 100 samples in the cohort and 10 batches were created, each containing DNA from 10 samples. This ensured that each batch contained DNA from samples with numbers starting with 0 [#0*], 1 [#1*], 2 [#2*], etc. These batches were termed row batches due to their orientation in the matrix (Fig. 1B, Additional file 1: Fig. S1) and were named using numbers; 0 to 9. The same process was repeated batching the same 100 samples again with 10 batches of 10 samples each. Only now, batches contained DNA from samples with numbers ending in 0 [#*0], 1 [#*1], 2 [#*2], etc. These batches were termed column batches and named using letters; A to J (Fig. 1A, Additional file 1: Fig. S1).

In this way, each sample was uniquely represented by exactly one row-batch and one column-batch and this yielded a matrix of 10 × 10 batches. In a matrix, each intersection represents a sample, e.g., the intersection of column batch H and row batch 4 corresponds to sample/individual #H4 (Fig. 1C, Additional file 1: Fig. S2). Finally, each of the 20 batches was sequenced to a target coverage of 2000X using Illumina’s TruSight Hereditary Cancer Panel covering (113-gene panel, Additional file 1: Table S1).

Identical methods were used to batch and sequence the validation cohort, yet, in order to also test scalability, four additional stand-alone batches, separate from the explorative and validation DoBSeq matrices, were constructed. Here, genomic DNA from Guthrie cards was batched using samples #00–#23, #00–#47, #00–#71, and #00–#95 from the explorative cohort (Additional file 1: Fig. S3). Target average coverage was 2000X per batch, which yielded proportionally lower per sample/allele coverage in the larger batches (Table 1).

**Table 1:** Results from the four stand-alone batches represented by one row each. Shows the mean target coverage per *n* (X), the expected variant allele frequency (VAF) for true heterozygous variants, unfiltered (raw) variant calls, loss-of-function or reported pathogenic (LoF/P) calls, as well as the number and VAF of true calls and known false calls

*n* in batch	Mean X	Expected VAF	Raw var. calls [mean VAF]	Raw LoF/P var. calls [mean VAF]	Matched LoF/P var. calls [mean VAF] (sensitivity)	False matches [mean VAF] to unique known var. of patients not in batch
24	83	2.08%	353,182 [0.21%]	29,415 [0.18%]	9 [1.80%] out of 9 (100%)	92 [0.26%] out of 453 (20%)
48	42	1.04%	295,492 [0.24%]	24,772 [0.20%]	17 [0.94%] out of 17 (100%)	61 [0.27%] out of 311 (20%)
72	28	0.69%	299,307 [0.19%]	25,210 [0.15%]	25 [0.63%] out of 26 (96%)	38 [0.22%] out of 168 (23%)
96	21	0.52%	287,905 [0.22%]	24,118 [0.18%]	31 [0.51%] out of 31 (100%)	2 [0.15%] out of 16 (13%)

### Variant calling and benchmarking

In the explorative cohort, unfiltered variants called in the 20 batches were immediately annotated using individual variant call data from the WGS truth-set. Hence, the analysis of variants found on DobSeq data in the explorative cohort was unblinded. Variants were called as unique in the explorative matrix by using an iterative method which accounted for sequencing noise levels (Additional file 1: Fig. S4). For each variant a uniqueness score (pinning the variant to an individual) was calculated with an aim of having perfect predictive values, while also leaving a margin for error/data variation (Additional file 1: Fig. S5). Lastly, variants pinnable to a specific patient were assessed for call quality or confidence. A confidence score of up to 100% was calculated using a weighted combination of 1) the observed mean VAF’s proximity to the theoretical allele contribution and 2) uniqueness score (excess uniqueness score above the call threshold). Variants were grouped as no, low, medium or high confidence, when the confidence score was <24.99%, 25.00% to 49.99%, 50.00% to 74.99%, or above 75.00%, respectively. The parameter thresholds were a product of empirical testing in the explorative cohort. Details are available in the supplemental materials.

Following the results of the explorative cohort, we ran a second blinded, but otherwise identical, experiment in the validation cohort (100 new patients). The analysis was blinded to the WGS data, and investigators were thus unaware of which patients carried genotypes of interest. Variant calling of the DoBSeq data was done precisely as dictated by the parameters determined by the explorative cohort. Then, after DoBSeq data analysis was completed using the specified cut-offs and the results were shared with an internal unblinded researcher, data was finally annotated with WGS data, and benchmarking analysis was conducted by comparing DoBSeq and WGS output (Additional file 1: Fig. S5).

### Cost estimates

Cost estimates were calculated using freely available list prices of sequencing flow cells and reagents. The commercial gene panel (Additional file 1: Table S1) was set at the price actually paid as part of this study. All cost estimates were based on yields listed by the manufacturers and were not corrected for data loss. Details are available in the supplementary materials.

### Statistics

R package EpiR (version 2.0.40) was used for computing true and apparent prevalence, sensitivity, specificity, positive and negative predictive values, and positive and negative likelihood ratios as well as exact binomial confidence limits based on count data in a 2 by 2 table format.

## Results

### Explorative cohort

We selected an explorative cohort (EC) of 100 participants, enriched for germline loss-of-function/known pathogenic (LoF/P) variants, among childhood cancer patients with WGS data available. WGS identified 31 high-confidence LoF/P variants [52% frameshift, 29% nonsense, 13% missense, and 6% splice] that were both unique to the cohort and covered by a commercial 113-gene panel (Fig. 1, Additional file 2: Table S4).

From each of the 100 EC participants, DNA was extracted from neonatal blood spots and split in two aliquots (Additional file 2: Table S6). Each of the two aliquots was then batched with DNA aliquots from nine other participants, according to a 10 by 10 matrix of all EC participants (Additional file 1: Fig. S1, Fig. S2). In this way, DNA aliquots from two participants were never batched together more than once, and each participant was therefore represented in a unique batch combination of one column and one row in the matrix (Fig. 1A–C).

The 113-gene panel was employed for sequencing of all 20 batches. Using WGS data to determine true variants, we optimized a bioinformatic approach for pinpointing variants from the gene panel to specific individuals in the matrix by cross-referencing unique variants in row and column batches. Final filtering parameters were determined empirically (Additional file 1: Fig. S4, Fig. S5) and identified 575 cohort-unique variants in the EC of which 35 were LoF/P (Additional file 1: Fig. S6, Additional file 2: Table S4). Of these, 89% were classified as being either high (27) or medium (4) confidence, and were considered positive, while the remaining 11% were classified as low (2) or no (2) confidence and were considered negative (Fig. 1C).

The 31 LoF/P variants called by DoBSeq had complete (100%) patient-specific overlap with the 31 LoF/P variants identified by the gold standard WGS data. Thus, using the filtering and confidence-scoring parameters determined in the EC analysis, DoBSeq showed 100% (95% CI 0.89–1.00) sensitivity and 100% (95% CI 0.95–1.00) specificity for the identification of cohort-unique LoF/P variants found by individual WGS (Fig. 1).

### Validation cohort

Next, we tested replicability in a validation cohort (VC) of 100 patients again selected from the childhood cancer cohort, but with no patients overlapping with the EC. Employing identical methods, we undertook variant calling using only the exact parameters determined in the EC analysis (Additional file 1: Fig. S5, Additional file 2: Table S6). All investigators remained blind to both patient identity and individual WGS data until the VC analysis was complete and LoF/P variants were reported (Fig. 1D–F).

Using only the predetermined parameters, DoBSeq called 537 cohort-unique variants in the VC of which 9 were LoF/P (Additional file 1: Fig. S7, Additional file 2: Table S4). Seven variants were of high (6) or medium (1) confidence and were considered positive, while two were of no confidence and were considered negative (Fig. 1F). The seven LoF/P variants that were positive on DoBSeq were reported to unblinded investigators and compared to LoF/P variants found by individual WGS data. Again, all 7 LoF/P variants found using DoBSeq had complete (100%), patient-specific overlap with the 7 LoF/P variants identified in the WGS data [43% frameshift, 29% nonsense, 14% missense, and 14% splice]. Thus, DoBSeq maintained 100% (95% CI 0.59–1.00) sensitivity and 100% (95% CI 0.96–1.00) specificity for the identification of cohort-unique LoF/P variants found with individual WGS (Fig. 1).

Combining results from both the explorative and the validation cohorts yielded a positive predictive value of having a cohort-unique LoF/P variant found on DoBSeq of 100% (95% CI 0.91–1.00) and negative predictive value of 100% (95% CI 0.98–1.00).

**Fig. 2:**
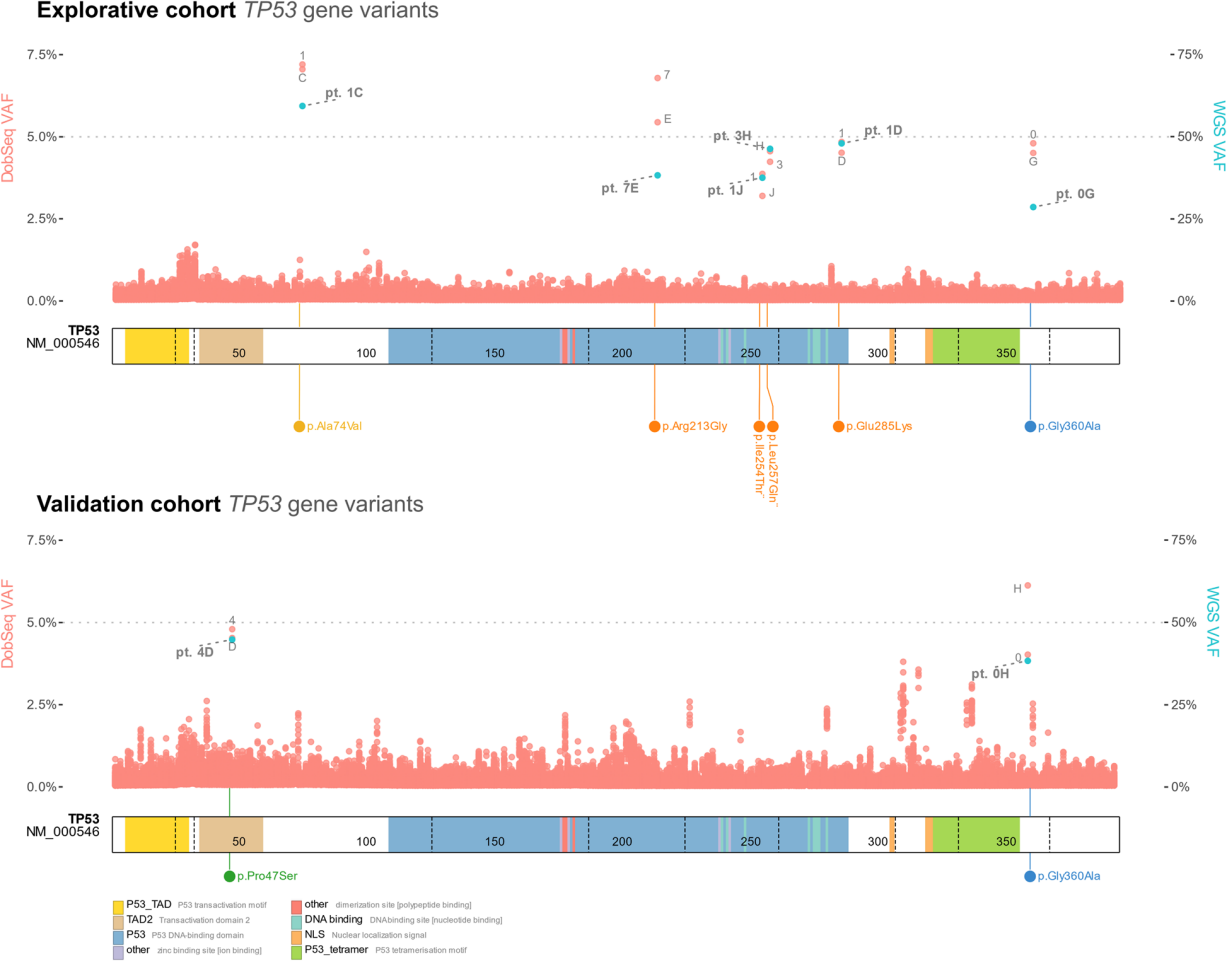
Reidentifying *TP53* variants with double batched sequencing (DoBSeq). Upper plot: Illustrates all non-synonymous coding variants in the *TP53* gene called without any filtering, i.e., includes all low-coverage and low-confidence calls, showing DoBSeq variants found in batches from the explorative cohort (EC) in red and whole genome sequencing (WGS) variants found in the EC in teal. The *x*-axis shows the canonical *TP53* protein product, with numbers indicating codon number and dotted lines indicating exonic borders. The *y*-axis shows variant allele frequency (VAF) for DoBSeq on the left and WGS on the right. The dotted line indicates the theoretical VAF for true heterozygous variants. On the *x*-axis, the variants found on WGS and reidentified with DoBSeq are indicated by lollipop markers with colors corresponding to ClinVar classifications of likely pathogenic (orange), variant of unknown significance (yellow), likely benign (blue), and benign (green). A marker (¨) was added to the protein change when the variant did not have a ClinVar classification; here color indicates in-house classification. A common polymorphism (p.Pro72Arg) found in 86 alleles was filtered out for the sake of clarity. *Interpretation:* A steady level of sequencing noise runs along the low end of the *y*-axis with individual sets of batches rising up towards or slightly above the expected VAF. This signal allows for pinpointing to individuals. Lower plot: As above; for the validation cohort. A common polymorphism (p.Pro72Arg) found in 92 alleles was filtered out for the sake of clarity

Focusing on *TP53*, a single gene of particular interest, we reidentified all 9 cohort-unique variants of any classification across both cohorts, including two variants [p.Ile254Thr and p.Leu257Gln] not reported as pathogenic in ClinVar, but immediately classified as such due to the location in the DNA binding domain and other phenotype or functional data. The validation cohort only revealed the anticipated non-pathogenic variants (Fig. 2).

### Scalability

**Fig. 3:**
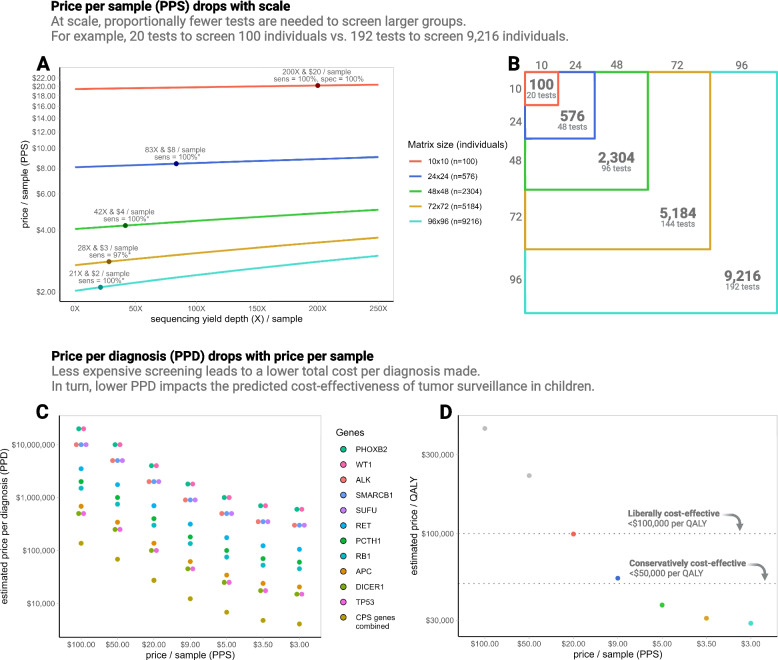
Illustrates scalability and estimated economic impact of lowered price per sample (PPS). **A** The PPS stratified by the size of the matrix, with line colors corresponding to those of the text and matrices in panel **B**. The *x*-axis represents sequencing yield in the targeted area, under a conservative assumption of 50% quality control data loss. The logarithmic *y*-axis shows the raw estimated price of sequencing the samples from a single individual. The dots represent empirical data from single batched sequencing at the specified batch sizes, including the sensitivity for loss-of-function and/or known pathogenic (LoF/P) variant detection. **B** Population size and number of tests needed to screen every individual using the double batched sequencing (DoBSeq) method. **C** The price per diagnosis (PPD, i.e., LoF/P variant detection) as influenced by PPS for 11 selected childhood cancer predisposition syndrome (CPS) genes corresponding to those investigated by Yeh et. al .[16], as well as all 11 genes combined. Legend is ordered by CPS prevalence. Prevalence is based on the best available evidence. **D** Based on the economic model by Yeh et al .[16] this graph illustrates the cost per quality-adjusted life-year (QALY) gained by tumor surveillance (within the 11 CPSs in panel **C**) at decreasing PPS. The dotted lines delineate screening costs that are cost-prohibitive, cost-effective at a liberal cut-off of $100k per QALY, and cost-effective at a conservative cut-off of $50k per QALY, respectively. Colored dots correspond to PPS at selected matrix sizes (see panel **B**)

Our first secondary endpoint was to test the sensitivity for detection of LoF/P variants when sequencing stand-alone batches of neonatal blood spot DNA including 24, 48, 72, and 96 individuals from the EC (Fig. 3B, Additional file 1: Fig. S3). The total coverage (X) per batch was kept constant, resulting in coverages per sample that were proportionally lower than the 200X achieved in the EC and VC. Across the scaled batches, expected LoF/P variants were detected at sensitivities ranging from 96 to 100%. Even at an average of 10X per allele (in the 96-sample stand-alone batch), 31 of 31 LoF/P variants were detected, resulting in a sensitivity that remained 100% (Fig. 3A, Table 1). Because the scaled batches were run as stand-alones, variants could not be pinpointed to individuals, which meant that specificity could not be meaningfully calculated. As remarked above, several false positive variants were called in individual batches, and these were only recognized as false when they were not doubly detected in DoBSeq. Yet, reassuringly, true positive variants showed higher VAFs than false positive variants, and the VAFs were very near the expected per-allele contribution (Table 1, Additional file 1: Fig. S9). Importantly, due to a combination of lower sequencing yield and lower contribution of each allele, false variants became increasingly difficult to distinguish from those known to be true (Additional file 1:Fig. S10).

At scale, the number of library preparations needed, and consequently cost, drops exponentially. This results in DoBSeq becoming increasingly cost-effective the more samples are sequenced in a matrix at once. We estimated costs per sample using various flow cells (Table 2) at different matrix sizes and target coverages (Table 3). Using the same 113-gene panel throughout, this yielded an estimated cost per sample as low as 2 USD, when a matrix of 96 times 96 (9216 individuals) was considered. Our calculations also reveal that even using a relatively small panel (covering 403 kilobases), large flow cells are required due to the high number of samples, further increasing the sequencing cost-effectiveness.

**Table 2:** Sequencing cost estimates at increasing matrix sizes using four commercial systems. Four commercial sequencers/flow cells used for sequencing cost estimates in Table 3

System and flow cell	Read size (paired-end)	# of reads	Output (Gbp)	Price (USD)	Cost/Gbp (USD)	Cost/1X panel coverage (USD)
NextSeq 500 Mid Output Kit v2 (300 cycles)	150 × 2	400M	120	5785 USD	48.21 USD	0.019 USD
NovaSeq 6000 SP Reagent Kit v1.5 (300 cycles)	150 × 2	800M	240	5100 USD	21.25 USD	0.009 USD
NovaSeq 6000 S2 Reagent Kit v1.5 (300 cycles)	150 × 2	4B	1200	12,150 USD	10.13 USD	0.004 USD
NovaSeq 6000 S4 Reagent Kit v1.5 (300 cycles)	150 × 2	10B	3000	17,700 USD	5.90 USD	0.002 USD

*Gbp* gigabasepair, *M* million, *B* billion

**Table 3:** Sequencing cost estimates at increasing matrix sizes using four commercial systems. Estimated raw sequencing cost using flow cells and list prices in Table 2 and calculated for conventional individual sequencing and double-batched sequencing at increasing scale; shown at four different target coverages. The estimates are all based on the 113-gene panel. Experiments indicate the number of full setups that can be run on flow cell

		100X panel coverage/n			200X panel coverage/n			400X panel coverage/n			800X panel coverage/n		
Setup	System (flow cell)	Cost/*n*	Cost prop.	Experiments	Cost/*n*	Cost prop.	Experiments	Cost/*n*	Cost prop.	Experiments	Cost/*n*	Cost prop.	Experiments
*n* = 1 (individual)	NextSeq (Mid Output)	104 USD	ref.	*n*=2978	106 USD	ref.	*n*=1489	110 USD	ref.	*n*=744	118 USD	ref.	*n*=372
	NovaSeq (SP)	103 USD	ref.	*n*=5955	104 USD	ref.	*n*=2978	106 USD	ref.	*n*=1489	109 USD	ref.	*n*=744
	NovaSeq (S2)	103 USD	ref.	*n*=29,777	103 USD	ref.	*n*=14,888	104 USD	ref.	*n*=7444	106 USD	ref.	*n*=3722
	NovaSeq (S4)	103 USD	ref.	*n*=74,442	103 USD	ref.	*n*=37,221	103 USD	ref.	*n*=18,610	104 USD	ref.	*n*=9,305
*n* = 100 (10×10 matrix)	NextSeq (Mid Output)	22 USD	21%	*n*=30	24 USD	23%	*n*=15	28 USD	26%	*n*=7	36 USD	31%	*n*=4
	NovaSeq (SP)	21 USD	21%	*n*=60	22 USD	21%	*n*=30	24 USD	23%	*n*=15	27 USD	25%	*n*=7
	NovaSeq (S2)	21 USD	20%	*n*=298	21 USD	21%	*n*=149	22 USD	21%	*n*=74	24 USD	22%	*n*=37
	NovaSeq (S4)	21 USD	20%	*n*=744	21 USD	20%	*n*=372	21 USD	21%	*n*=186	22 USD	21%	*n*=93
*n* = 576 (24×24 matrix)	NextSeq (Mid Output)	10 USD	10%	*n*=5	12 USD	12%	*n*=3	16 USD	15%	*n*=1	24 USD	20%	*n*=1
	NovaSeq (SP)	9 USD	9%	*n*=10	10 USD	10%	*n*=5	12 USD	11%	*n*=3	15 USD	14%	*n*=1
	NovaSeq (S2)	9 USD	9%	*n*=52	9 USD	9%	*n*=26	10 USD	10%	*n*=13	12 USD	11%	*n*=6
	NovaSeq (S4)	9 USD	9%	*n*=129	9 USD	9%	*n*=65	9 USD	9%	*n*=32	10 USD	10%	*n*=16
*n* = 2304 (48×48 matrix)	NextSeq (Mid Output)	6 USD	6%	*n*=1	8 USD	8%	*n*=1	12 USD	11%	*n*=0	20 USD	17%	*n*=0
	NovaSeq (SP)	5 USD	5%	*n*=3	6 USD	6%	*n*=1	8 USD	7%	*n*=1	11 USD	10%	*n*=0
	NovaSeq (S2)	5 USD	5%	*n*=13	5 USD	5%	*n*=6	6 USD	6%	*n*=3	8 USD	7%	*n*=2
	NovaSeq (S4)	5 USD	4%	*n*=32	5 USD	5%	*n*=16	5 USD	5%	*n*=8	6 USD	6%	*n*=4
*n* = 5184 (72×72 matrix)	NextSeq (Mid Output)	5 USD	5%	*n*=1	7 USD	6%	*n*=0	11 USD	10%	*n*=0	18 USD	16%	*n*=0
	NovaSeq (SP)	4 USD	4%	*n*=1	5 USD	4%	*n*=1	6 USD	6%	*n*=0	10 USD	9%	*n*=0
	NovaSeq (S2)	3 USD	3%	*n*=6	4 USD	4%	*n*=3	4 USD	4%	*n*=1	6 USD	6%	*n*=1
	NovaSeq (S4)	3 USD	3%	*n*=14	3 USD	3%	*n*=7	4 USD	4%	*n*=4	5 USD	5%	*n*=2
*n* = 9216 (96×96 matrix)	NextSeq (Mid Output)	4 USD	4%	*n*=0	6 USD	6%	*n*=0	10 USD	9%	*n*=0	18 USD	15%	*n*=0
	NovaSeq (SP)	3 USD	3%	*n*=1	4 USD	4%	*n*=0	6 USD	5%	*n*=0	9 USD	8%	*n*=0
	NovaSeq (S2)	3 USD	2%	*n*=3	3 USD	3%	*n*=2	4 USD	4%	*n*=1	5 USD	5%	*n*=0
	NovaSeq (S4)	2 USD	2%	*n*=8	3 USD	3%	*n*=4	3 USD	3%	*n*=2	4 USD	4%	*n*=1

*prop.* proportion [using individual sequencing cost as reference (ref.)]

### All unique variants

Another secondary endpoint of this study was to test DoBSeq’s ability to find any variant unique to the cohort, regardless of mutation ontology or pathogenicity. As further detailed in the supplementary materials, DoBSeq correctly identified and pinpointed carriers of high confidence unique variants as determined by WGS data at a rate of 94% (1034/1097) without adjustments, yielding a positive predictive value of 98.95% (95% CI 98.93–98.96%) (Additional file 1: Table S2). This rose to 97% (1034/1065) when discounting non-coding and pseudogenic false negative variant calls (Additional file 2: Table S5). True negatives are not meaningful to consider as any one loci identified by both WGS and DoBSeq as wildtype or reference may be considered true negatives and these count in the hundreds of thousands. As a whole, our findings suggest that DoBSeq’s performance is non-inferior to variant-calling concordance of conventional individual sequencing cross-platform comparisons [17].

## Discussion

We demonstrate that the DoBSeq approach to mass genetic screening for rare disease-causing variants is a reliable and cost-effective method, which is likely to be highly scalable and hence applicable to population screening. The high performance and ability to directly pinpoint carriers of genetic variants are a product of the repeated sequencing of the same individual in two separate batches. Essentially, our results fully support that DoBSeq applied on large cohorts can directly identify single individuals with rare variants. The identified carriers may then be confirmed using conventional sequencing, after which clinical reporting could take place in adherence to existing best practices [18, 19]. To eliminate interpretation biases in the present analysis, we purposefully excluded variants that were internally classified as pathogenic (Fig. 2).

Surprisingly, our data showed that false positive LoF/P variants with passable quality parameters were widespread when the sequencing results from stand-alone batches were assessed in isolation (Fig. 1A–B, D–E). However, by filtering variants to those seen in a combination of one column and one row batch, i.e., appearing twice in two different batches, we could clearly distinguish between true and false positive variants. This suggests that, using our method, single batched sequencing (in which carriers cannot be pinpointed) would require copious individual resequencing. Considering the high number of RDs in the general population, the pursuit of just the true positive variants could become costly if dozens or even hundreds of genes were screened simultaneously, with each finding requiring resequencing of all individuals in the stand-alone batch. Based on our data, false positives in stand-alone batches were common, however, our design was not specifically optimized with highly accurate variant calling in stand-alone batches in mind, hence the resequencing needed in pursuit of variant which would ultimately turn out to be false, could not be reliably assessed based on our data.

Currently, well over 400 gene-disease pairs are considered highly actionable in childhood with respect to age-of-onset and/or timing of intervention, and at least an additional 25 are highly actionable in adulthood [5]. Each of these conditions may be considered viable candidates for population screening, yet, primarily due to cost restrictions, few are routinely screened for in any healthcare system and no screening currently uses genomics up-front [8, 20].

A recent simulation model evaluated the cost-effectiveness of universal screening for a panel including 11 pediatric CPS genes [16], which fully overlapped with the 113-gene panel used in our study. Because RDs, such as pediatric CPSs, by definition have a very low prevalence, the number-needed-to-diagnose (NND) in an unselected cohort is high. For individual rare conditions, such as *WT1*-related disorders, the price per diagnosis (PPD) at the estimated cost of doing a single genetic test likely exceeds $10 million (Fig. 3C). Of course, by using panels, several conditions can be screened for simultaneously, lowering the NND for any one condition, yet, for the 11 pediatric CPS genes, Yeh et al. [16] conclude that at the current best price per sample (PPS) of $55, tumor surveillance strategies are cost-prohibitive ($244,860 per life-year gained). However, at a PPS of $20, surveillance approaches liberal cost-effectiveness (<$100,000 per life-year gained) (Fig. 3D). Practically, the PPD must be added to the isolated cost of the tumor surveillance strategy, making it increasingly likely that any given treatment, which is cost-effective in isolation, will remain cost-effective as PPD is reduced. According to the models developed by Yeh et al., the tumor surveillance strategies and treatments available for the 11 conditions they studied will be even conservatively cost-effective (<$50,000 per life-year gained) if PPS drops below $8, which we demonstrate to be highly possible when DoBSeq is run at scale (Fig. 3D).

DoBSeq has the potential to advance genetically based risk stratification, and the cost-effectiveness of doing so, for hundreds of actionable RDs caused by rare genetic variants. This is by no means limited to DNA samples from neonatal blood spots. Several studies have investigated the cost-effectiveness of population screening for adult CPSs [21–25], and one such study [25], investigating the impact of PPS on universal adult screening for *BRCA1/2* and MMR genes, found it to be conservatively cost-effective even at PPS exceeding $1000. Lowering PPS to those estimated for DoBSeq running at scale could lead to prices per life-year gained that approach cost-saving (Additional file 1: Fig. S11, Fig. S12). Most adult CPSs are believed to be undiagnosed, and therefore population-based cancer screening stands to improve with genetically informed precision prevention [24].

The promising aspects of DoBSeq must be viewed in light of some limitations of the method and of this study. If multiple individuals in the same DoBSeq matrix carry exactly the same variant, it may not be possible to pinpoint the carriers directly. In this event, however, the number of possible carriers will be limited to a small group amenable for individual (re)sequencing. For instance, if two individuals carried the same variant, (re)sequencing of four individuals would be required to identify the two true carriers. Still, genetic heterogeneity, meaning that a specific disease may be caused by a myriad of distinct genetic defects, generally makes multiple carriers of molecularly identical variants in the same matrix astronomically unlikely. This was also found in our study, e.g., for *TP53* where four distinct variants all caused the same condition (Li-Fraumeni Syndrome) associated with a high risk of childhood cancers [26] (Fig. 2).

In some populations, pathogenic founder variants causing genetic diseases that are rare globally may be common enough to make person-specific variant detection using DoBSeq challenging. Nevertheless, founder variants are virtually always known and well-studied in the populations that carry them at high frequencies, and therefore such variants may be better suited for bespoke detection methods, such as was done for the Brazilian *TP53* founder variant, which was neonatally, and highly cost-effectively, screened for using a direct restriction fragment length polymorphism assay in the Paraná region of Brazil [27]. Conversely, DoBSeq’s use lies in identifying the highly heterogeneous mutational spectrum of non-founder, i.e., personal, variants, which cause the vast majority of serious genetic diseases in humans. Theoretically, the presented method should provide conclusive results for virtually all loci where variants have an allele frequency of 0.001% or lower (Additional file 1: Table S3).

Another possible limitation was that prior to batching, our method harmonized DNA concentration across all samples, which added a minor cost for each sample. At scale, this may impact overall cost-effectiveness estimates and we did not test whether removing this step impacted the performance of DoBSeq. Lastly, structural variants, known to cause around 10% of RD [28], were not investigated as part of the present study.

Lately, combinatorial pooling strategies, like DoBSeq, have received increased attention as a theoretical alternative to the extensive and costly population screening for the SARS-CoV-2 virus [29]. While the methods appear similar, a crucial difference is that a heritable disease may be caused by a myriad of distinct genetic variants, whereas SARS-CoV-2 tests, by and large, are positive in the same way.

Finally, we would be remiss not to mention that the implementation of mass genetic screening of healthy neonates, children, and/or adults, precipitates critical ethical considerations [30]. Ethics is not a focus of our current study, however, it is important to note that our method detects rare variants only, thus limiting the amount of personal genetic data obtained per individual. Moreover, if our method is developed further, the low price per sample may extend accessibility in low- and middle-income countries, making it more equitable than current individual NGS-based methods.

## Conclusions

Our DoBSeq method reliably detects rare pathogenic germline variants in populations at single nucleotide resolution — while reducing costs up to 20-fold. Our findings indicate that the approach is highly scalable and may easily be incorporated into existing screening infrastructures based on the collection of standard blood spot samples sequenced with standard NGS hardware. Consequently, DoBSeq could pave the way for cost-effective, at-scale, NGS-based population screening, and open up possibilities for efficient studies of large, unselected cohorts.

## Supplementary Information

**Additional file 1.** Containing supplementary Methods and Results, including additional figures and tables; **Fig. S1-S13** and **Table S1-S3**.

**Additional file 2. **Containing a workbook with three sheets of supplementary tables, **Table S4-S6.** The first sheet, “LoFP_variants”, contains details on unique loss-of-function and known pathogenic variants in the explorative and validation cohorts. The second sheet, “False_negative_variants”, contains details on false negative variants across all unique variants found in the explorative and validation cohorts. The third sheet, “DNA_yield, contains details on DNA extraction yield from Guthrie cards for both cohorts. For all tables; ID is formed by a letter (corresponding to the column batch the patient is in) and a number (corresponding to the row batch the patient is in). For first and second sheet; HGVS, Human Genome Variation Society (variant nomenclature). VAF, variant allele frequency, alt, alternative variant allele number, X, total read depth for locus, and SB, strand bias, are given for whole genome sequencing (WGS), column batch sequencing and row batch sequencing data.

## Data Availability

All publicly available data has been included in the manuscript and supplemental materials. The whole genome and double-batched panel-based sequencing generated and/or analyzed during the current study are not publicly available because the data is subject to GDPR and is legally considered person-identifiable, but are available from the corresponding author on reasonable request. Full data access can be facilitated by the corresponding author, with time to response to request of 30 days. Access will require a data sharing agreement granted by Danish authorities — a process that may take more than 30 days and is dependent on the data storage facilities of the intended host. Even if full data access is granted, the data will still be restricted in use as in accordance with GDPR rules and related privacy and ethical/legal considerations. Data access will also be subject to an internal discussion regarding authorship and ownership of any resultant scientific work.

## Abbreviations

CI: Confidence interval
CPS: Cancer predisposition syndrome
DNA: Deoxyribonucleic acid
DoBSeq: Double batched sequencing
EC: Explorative cohort
LoF/P: Loss-of-function/known pathogenic
MMR: Mismatch repair
NGS: Next-generation sequencing
NND: Number needed to diagnose
PPD: Price per diagnosis
PPS: Price per sample
QALY: Quality-adjusted life-year
RD: Rare disease
SARS-CoV-2: Severe acute respiratory syndrome coronavirus 2
STAGING: Sequencing Tumor and Germline DNA - Implication and National Guidelines
VAF: Variant allele frequency
VC: Validation cohort
WGS: Whole genome sequencing
