# Supplementary material for: Combinatorial batching of DNA for ultralow-cost detection of pathogenic variants
Source: Genome Med. 2023 Mar 14;15:17. doi: 10.1186/s13073-023-01167-6 (PMC10013285; doi:10.1186/s13073-023-01167-6)
Supplement: Supplementary file 1 — Additional file 1. Containing supplementary Methods and Results, including additional figures and tables; Fig. S1-S13 and Table S1-S3. [file 13073_2023_1167_MOESM1_ESM.docx]

##

[Supplemental methods 2](#_Toc119158329)

[Endpoints 2](#_Toc119158330)

[Primary endpoint 2](#_Toc119158331)

[Secondary endpoint 2](#_Toc119158332)

[Cohorts 2](#_Toc119158333)

[National pediatric cancer cohort 2](#_Toc119158334)

[Explorative cohort 3](#_Toc119158335)

[Validation cohort 3](#_Toc119158336)

[Data 4](#_Toc119158337)

[Gene panel 4](#_Toc119158338)

[Gold standard: Whole-genome sequencing (WGS) of DNA from peripheral blood samples 5](#_Toc119158339)

[Double-Batched sequencing (DobSeq) of neonatal blood-spots 6](#_Toc119158340)

[Data analysis 9](#_Toc119158341)

[Statistics 13](#_Toc119158342)

[Ethical approvals 14](#_Toc119158343)

[Other supplemental data 15](#_Toc119158344)

[Filtering results 15](#_Toc119158345)

[All unique variants regardless of clinical relevance 17](#_Toc119158346)

[DNA extraction yields 18](#_Toc119158347)

[Theoretical cost savings 19](#_Toc119158348)

[Calculated cost savings 21](#_Toc119158349)

[Analysis of scaled batches 22](#_Toc119158350)

[Explorative cohort LoF/P variants 25](#_Toc119158351)

[Validation cohort LoF/P variants 26](#_Toc119158352)

[Cost-effectiveness: Adult CPSs 27](#_Toc119158353)

[References 30](#_Toc119158354)

## Supplemental methods

### Endpoints

#### Primary endpoint

The primary endpoint of this work was to establish a bioinformatic methodology, which could reliably call cohort-unique, clinically relevant single nucleotide variants (SNVs) in individuals based solely on DobSeq data when benchmarked against whole genome sequencing (WGS). Cohort-unique refers to a variant only observed exactly once in the cohort being studied. Furthermore, to avoid selection bias, we defined variants as clinically relevant when they had a consensus classification as either ‘pathogenic’ or ‘likely pathogenic’ in the external database ClinVar and/or the variant led to a loss-of-function (LoF) variant, here defined as any frameshift, nonsense, or splice donor/acceptor variants, in the canonical transcript of the gene.

#### Secondary endpoint

Evaluating DoBSeq’s scalability by testing detection rates of clinically relevant variants in batches with an increased number of patients and correspondingly lowered per-allele coverage. Evaluating DoBSeq’s ability to detect any cohort-unique variant regardless of ontology and pathogenicity.

### Cohorts

#### National pediatric cancer cohort

In the design of our explorative and validation cohorts used for testing the DoBSeq method, we used participants from a Danish childhood cancer genomics study called STAGING (Sequencing Tumor and Germline DNA - Implication and National Guidelines)[^1^](https://www.zotero.org/google-docs/?jJGMPC). All participants had cancer and were 17 years old or younger at the time of diagnosis. All included patients underwent germline WGS as part of this study.

#### Explorative cohort

We constructed a cohort of 100 patients based on the national pediatric cancer cohort. Part of the national cohort has been published elsewhere[^1^](https://www.zotero.org/google-docs/?pT3D4e). The explorative cohort is a subpopulation of 439 total participants, selected firstly for the availability of neonatal Guthrie cards (born in Denmark), and secondly for the presence of pathogenic germline variants in genes covered by the gene panel described below.

#### Validation cohort

We constructed a second cohort of 100 new patients based on the same national cohort, at a time when the prospective inclusion had accrued an additional 110 patients. The validation cohort is a subpopulation of 449 participants, selected for non-inclusion in the explorative cohort, but otherwise identically to the explorative cohort.

### Data

| 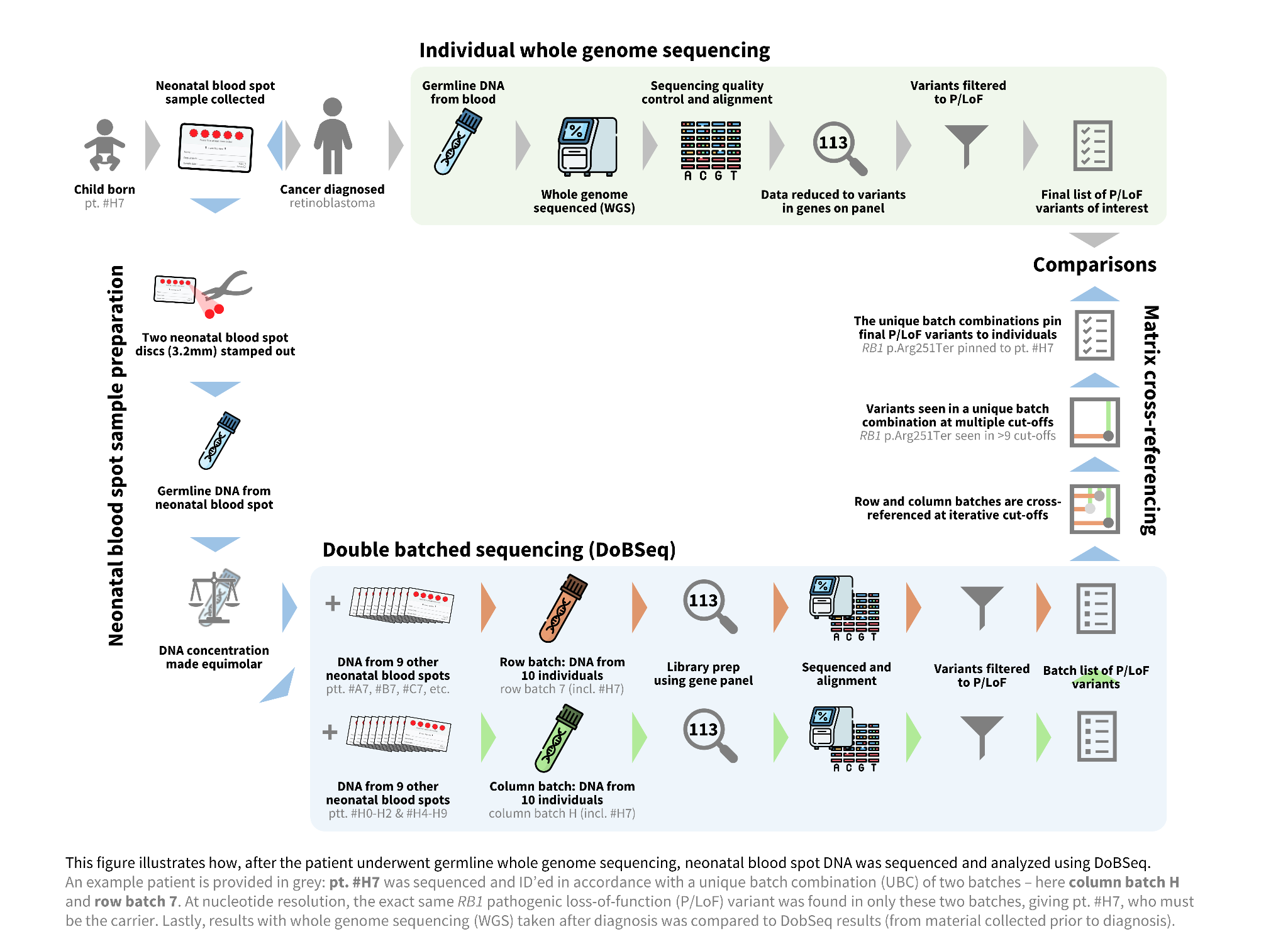 |
| --- |
| **Fig. S1**: This figure illustrates how, after the patient underwent germline whole genome sequencing, neonatal blood spot DNA was sequenced and analyzed using DoBSeq.  An example patient is provided in gray: **pt. #H7** was sequenced and ID’ed in accordance with a unique batch combination (UBC) of two batches – here **column batch H** and **row batch 7**. At nucleotide resolution, the exact same *RB1* pathogenic loss-of-function (P/LoF) variant was found in only these two batches, giving pt. #H7, who must be the carrier. Lastly, results with whole genome sequencing (WGS) taken after diagnosis were compared to DobSeq results (from material collected prior to diagnosis). |

#### Gene panel

For each of the 20 row-batches and 20 column-batches (10 row- and 10 column-batches in each of the explorative and validation cohorts) plus four larger additional batches, Illumina DNA Prep enrichment and library prep were performed using Illumina’s TruSight Hereditary Cancer Panel (<http://www.illumina.com/TruSightHereditaryCancer>) covering 113 genes associated with cancer predisposition (Table S1).

| *ACD* | *BRCA2* | *CTRC* | *FAM175A* | *FANCM* | *LZTR1* | *MUTYH* | *PMS2* | *RAD51C* | *SDHB* | *STK11* | *XPA* |
| --- | --- | --- | --- | --- | --- | --- | --- | --- | --- | --- | --- |
| *AIP* | *BRIP1* | *DDB2* | *FANCA* | *FH* | *MAX* | *NBN* | *POLD1* | *RAD51D* | *SDHC* | *SUFU* | *XPC* |
| *AKT1* | *CASR* | *DICER1* | *FANCB* | *FLCN* | *MEN1* | *NF1* | *POLE* | *RB1* | *SDHD* | *TERF2IP* | *XRCC2* |
| *APC* | *CDC73* | *DIS3L2* | *FANCC* | *GALNT12* | *MET* | *NF2* | *POT1* | *RECQL4* | *SLX4* | *TERT* |  |
| *ATM* | *CDH1* | *EPCAM* | *FANCD2* | *GATA2* | *MITF* | *NSD1* | *PRKAR1A* | *RET* | *SMAD4* | *TMEM127* |  |
| *BAP1* | *CDK4* | *ERCC1* | *FANCE* | *GPC3* | *MLH1* | *NTHL1* | *PTCH1* | *RHBDF2* | *SMARCA4* | *TP53* |  |
| *BARD1* | *CDKN1B* | *ERCC2* | *FANCF* | *GREM1* | *MRE11A* | *PALB2* | *PTEN* | *RINT1* | *SMARCB1* | *TSC1* |  |
| *BLM* | *CDKN2A* | *ERCC3* | *FANCG* | *HOXB13* | *MSH2* | *PDGFRA* | *RAD50* | *RUNX1* | *SMARCE1* | *TSC2* |  |
| *BMPR1A* | *CEBPA* | *ERCC4* | *FANCI* | *KIF1B* | *MSH3* | *PHOX2B* | *RAD51* | *SDHA* | *SPINK1* | *VHL* |  |
| *BRCA1* | *CHEK2* | *ERCC5* | *FANCL* | *KIT* | *MSH6* | *PIK3CA* | *RAD51B* | *SDHAF2* | *SPRED1* | *WT1* |  |

| **Table S1:** The 113 cancer predisposition genes covered by Illumina’s TruSight Hereditary Cancer Panel used for sequencing batches in the explorative and validation cohorts. |
| --- |

#### Gold standard: Whole-genome sequencing (WGS) of DNA from peripheral blood samples

All 200 participants in the explorative and validation cohorts had WGS data from germline DNA available at the outset of the study. Sequencing protocols have been published in detail elsewhere[^1^](https://www.zotero.org/google-docs/?omKRRa). Briefly, leukocytic DNA was extracted from peripheral blood samples and WGS was performed using the HiSeqX platform (Illumina, San Diego, CA, USA) with paired-end sequencing of 150bp reads and target 30X average coverage. For patients with hematologic malignancies, blood samples were drawn after remission or otherwise skin biopsies were obtained. Reads were mapped to the hg19 reference genome sequence (GRCh37.p13; RefSeq assembly accession GCF_000001405.25) using GATK version 3.8 or the DNAseq pipeline (Sentieon, San Jose, CA, USA). VarSeq software (version 2.2.0, Golden Helix, Bozeman, MT, USA) was used to annotate variants.

The WGS variant call files were subsetted to genomic areas corresponding to the panel (Table S1) using bcftools/1.10. This subset constituted the raw gold standard reference for single nucleotide germline variants present in the cohorts (Fig. S1).

#### Double-Batched sequencing (DobSeq) of neonatal blood-spots

All 200 participants in the explorative and validation cohorts had neonatal screening Guthrie cards (GC), i.e. dry blood spot sample (DBSS) on filter paper, available in the Danish Neonatal Screening Biobank (DNSB). GC are routinely collected in the first few days of life and residual materials are stored in the DNSB at negative 20 degrees Celsius, in the sequence in which they were originally received for NBS analyses. From each GC two 3.2 mm DBSS discs were stamped into a sample collection tube. DNA was extracted from both DBSS disks using the NEB Monarch Genomic DNA Extraction and Purification Kit (New England Biolabs, Ipswich, MA, USA).

DNA concentrations were measured using QuantIT kits optimized to the dsDNA/ssDNA ratio. DNA concentrations were then normalized to ensure equimolar contributions of DNA from each sample. Concentrations were matched to the protocol for the Illumina DNA Prep with enrichment platform. Samples in each of the two cohorts were randomly allocated with a number from #00 to #99. Two sets of 10 batches were compiled for each cohort:

1. Volumes equal to 10ng of genomic DNA were taken from each of the 100 samples in the cohort and 10 sets of 10 samples were pooled into batch tubes containing DNA from samples with numbers starting with 0 [#0*], 1 [#1*], 2 [#2*], etc. These batches were termed row batches due to their orientation in the matrix (Fig. S1) and were named using numbers; 0 to 9.
2. Step one was repeated, batching the same 100 samples again with 10 sets of 10 samples pooled into batch tubes now containing DNA from samples with numbers ending in 0 [#*0], 1 [#*1], 2 [#*2], etc. These batches were termed column batches and named using letters; A to J ( Fig. S1).

| 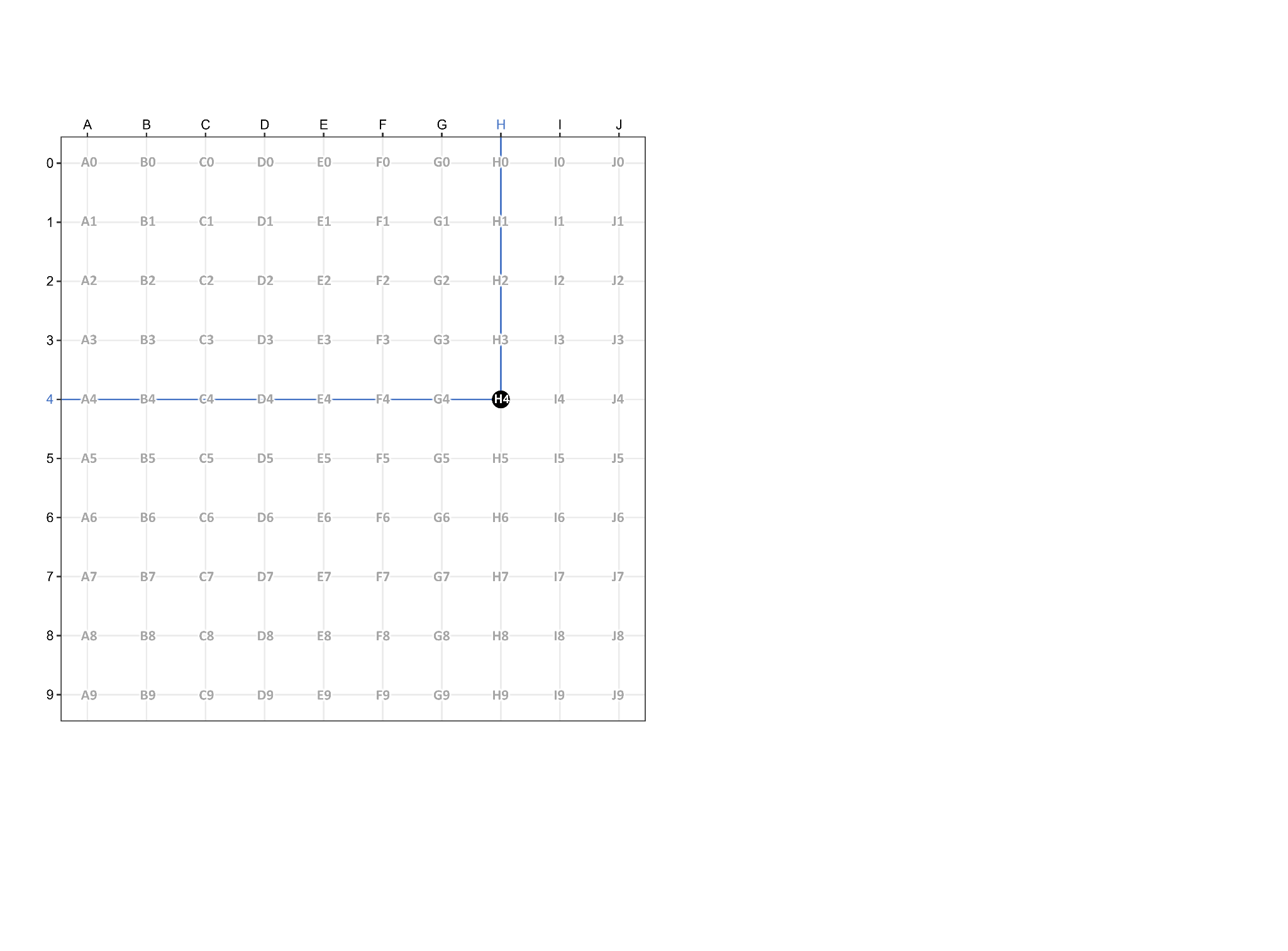 |
| --- |
| **Fig. S2:** The experimental structure of a 10 x 10 matrix highlighting the column batches along the x axis named with a letter from A to J, and row batches along the y axis named with a number from 0 to 9. Each individual or sample is present in a unique combination of one column and one row represented by the intersections. Individual or sample #H4 is pinpointed by being present in column batch H and row batch 4. |

Thus each sample is represented in exactly one row-batch and one column-batch. This double batching strategy yielded a matrix of 10 x 10 samples for each cohort. In these matrices, each intersection represents a sample, e.g. the intersection of column batch H and row batch 4 corresponds to sample/individual #H4 (Fig. S2).

In order to test scalability, four additional one-dimensional “scalability” batches, separate from the matrices, were constructed, containing 24, 48, 72 and 96 samples. Here, genomic DNA from DBSS was batched using samples #00 - #23, #00 - #47, #00 - #71, and #00 - #95 from the explorative cohort, respectively (Fig. S3).

For each of the 20 row-batches and 20 column-batches plus four larger additional batches, Illumina DNA Prep enrichment and library prep were performed using Illumina’s Hereditary Cancer Panel covering 113 genes. Following library preparation the batches were normalized, batched using equimolar DNA, then sequenced on NextSeq 500 platform using a High-Output 150bp paired-end sequencing kit and flow cell. Target average coverage was 2000X per batch, equal to 200X/100X per sample/allele in the 40 column/row batches, and 83X/42X per sample/allele, 42X/21X per sample/allele, 28X/14X per sample/allele, and 21X/10X per sample/allele, for the scaled batches containing 24, 48, 72, and 96 samples, respectively.

| 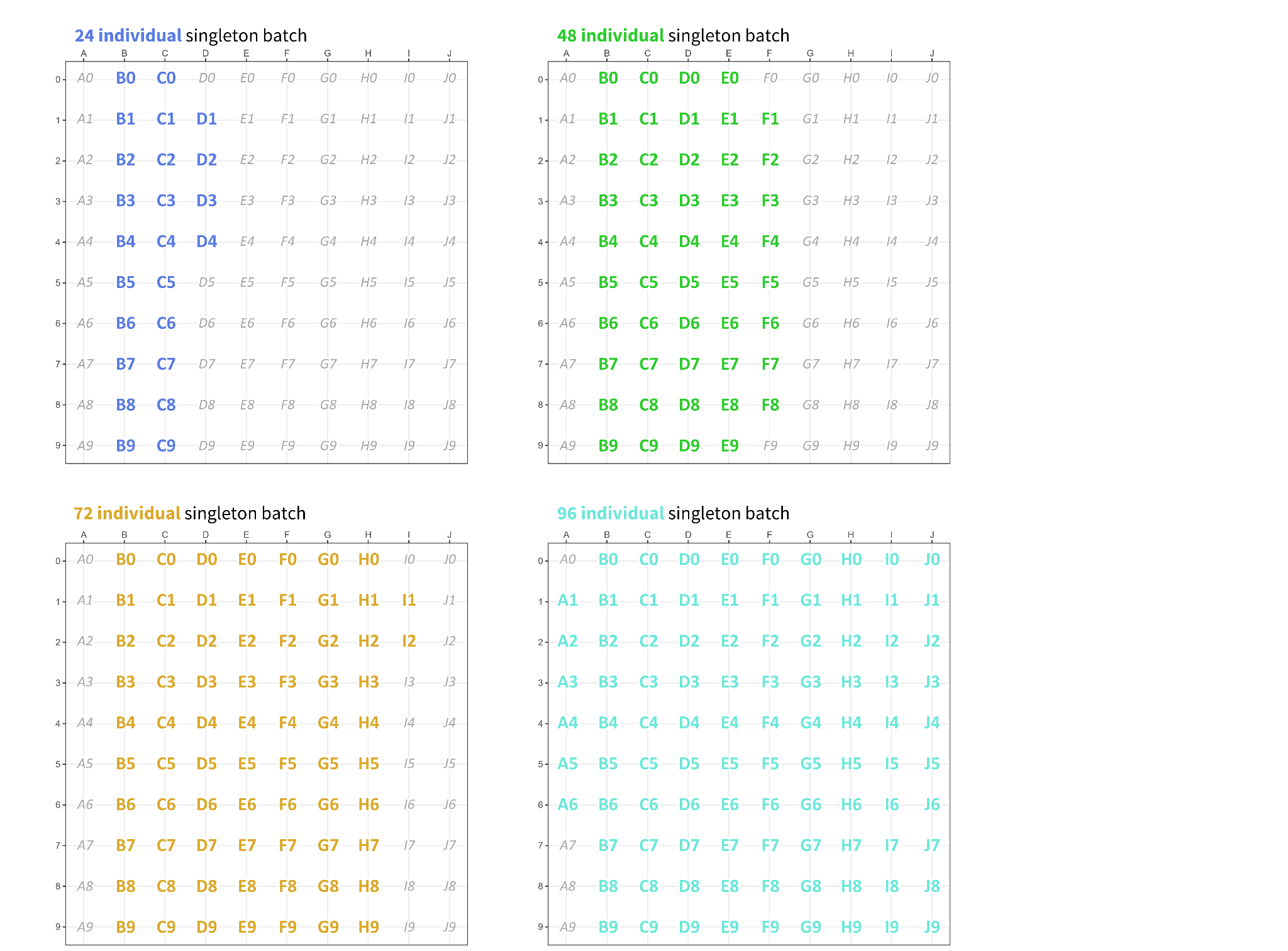 |
| --- |
| **Fig. S3:** Shows which individual, all from the explorative cohort, were in each of the scaled batches. |

### Data analysis

**Standard NGS pipeline**

Reads were mapped to GRCh37 (GCF_000001405.25) using BWA mem (v.0.7.16a-r1181), duplicated reads removed using picard (v.2.20.8-SNAPSHOT) and base quality scores recalibrated using GATK (v.4.0.0.0). Variants were called using Lofreq (v.2.1.0) with all filters disabled. VarSeq software (version 2.2.0, Golden Helix, Bozeman, MT, USA) was used to annotate variants.

**Defining variants of interest**

In order to limit our analysis to variants of interest, we defined variants as being clinically relevant by two criteria: 1) the loss-of-function (LoF) criteria [predicted to cause protein-truncation by being frameshift, nonsense or splice acceptor/donor variants] and/or 2) the reported pathogenic (P) criteria [having a consensus classification in ClinVar of pathogenic or likely pathogenic]. Variants in this category were labeled as LoF/P. This restrictive definition, based solely on *in silico* ontology and third-party variant classifications, aimed to exclude internal interpretation biases such as phenotype-driven pathogenicity scoring as phenotypes would not be available in a screening scenario.

**DoBSeq unique variant calling**

Data analysis was performed on R (4.1.0) using RStudio (version 1.2.5001) and employing the R metapackage Tidyverse (version 1.3.1).

Unfiltered variant call files from all 24 batches (explorative cohort matrix and scalability batches) were annotated with VarSeq software (version 2.2.0, Golden Helix, Bozeman, MT, USA) and loaded into R. With the intent of full navigability in setting appropriate parameters, the explorative matrix data were annotated using individual variant call data from the WGS truth-set, resulting in an explorative unblinded analysis. WGS data contain several sequencing artifacts, thus the following filters were applied to the total WGS data:

1. VAF equal to or higher than: **0.3**
2. Depth equal to or higher than: **15X**
3. Strand bias equal to or lower than: **20**
4. Multiallelic calls were excluded.

Variants were called as unique to the explorative or validation matrices using a bespoke iterative method explained in the following. Variants were considered unique when they arose in precisely one column batch and one row batch, i.e. in a unique batch combination (UBC). Due to inherent errors in NGS data, a variable level of false base calls, i.e. background noise, is expected. These lead to false positive calls only when the exact same variant is randomly seen in a UBC. Any one specified cut-off risks calling such false positive (FP) variants, because areas with a high tendency for noise may also randomly meet exactly the specified cut-off in a UBC - despite many other batches also calling the same variant, albeit, at levels just below the threshold. True variants in error-prone areas will only start showing UBC when they rise above a given threshold, while true variants in areas with low error rate will show a UBC at a very low or no cut-off. For this reason we used an iterative cut-off method:

1. 25 iterations of hard cut-offs were run:
   1. The first iteration called all variants with a UBC at an alternate allele count cut-off set to 1X.
   2. In each subsequent iteration, the alternate allele count cut-off was increased by 1X.
2. At each iteration, a uniqueness score of 1 was added to the variant if it was seen in a UBC at that iteration. All other variants got a score of 0. Thus a variant may receive a uniqueness score equal to, at most, the number of iterations run (the variant is seen in a UBC at every cut-off), and down to the lowest score, zero, for variants that are not called in a UBC in any iteration (Fig. S4).
3. Lastly, we assessed the performance of the uniqueness parameter in calling false and true variants (Fig. S5), and variants were filtered for being truly unique if they had a uniqueness score of 10 or higher.

| 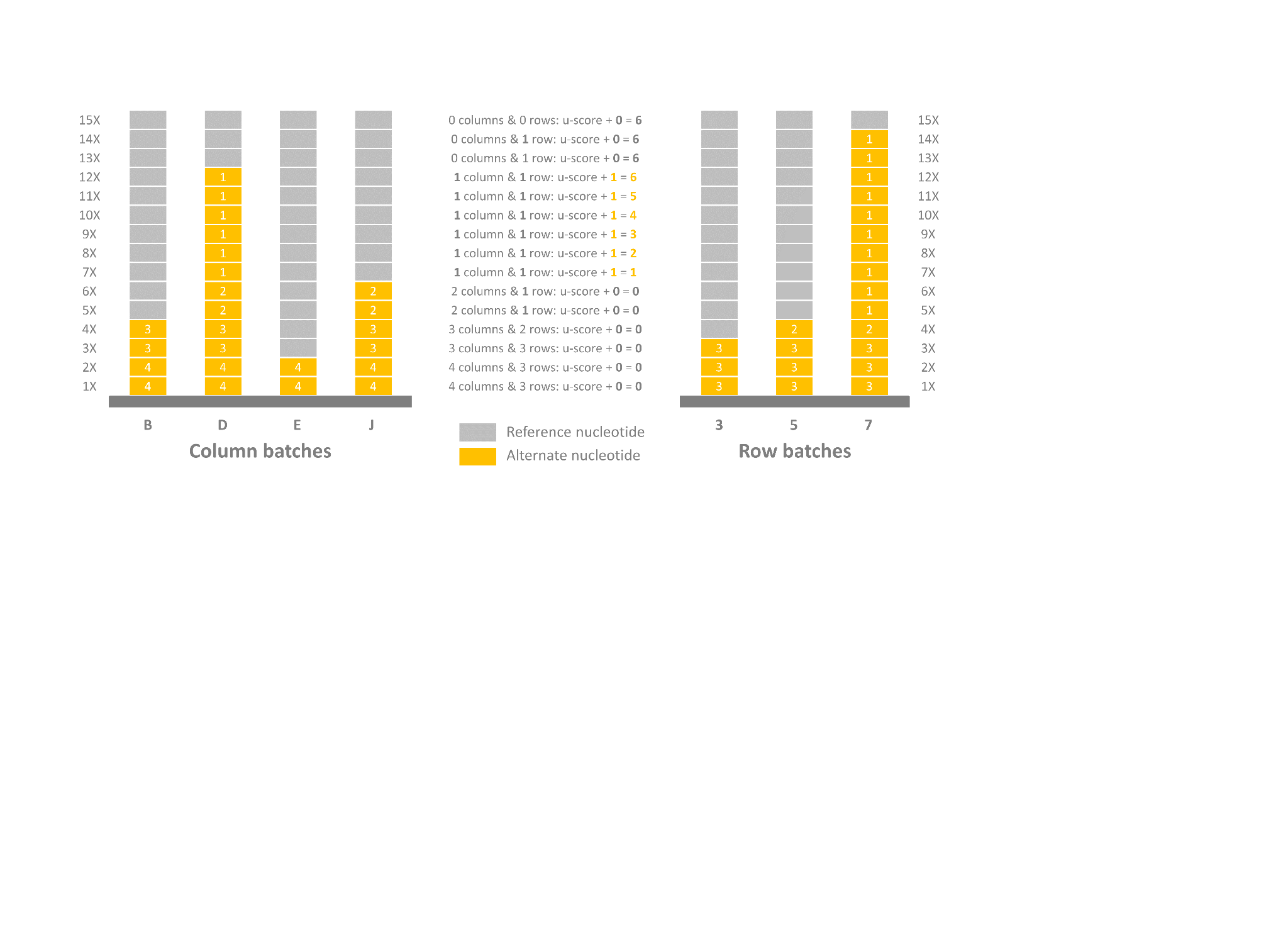 |
| --- |
| **Fig. S4:** A simplified illustration of how uniqueness score (u-score) was calculated. In this example, alternate (yellow) and reference (gray) base calls for a given specific variant are shown. The variant has been called in 4 column batches (left) and 3 row batches (right). Iterations of hard cut-offs check whether the variant can be pinned to a unique batch combination (UBC) at each iterative cut-off increment of 1X coverage. When a variant is called as unique at a cut-off it is given +1 u-score (middle), which occurs at cut-offs 7X through 12X yielding a u-score of 6, pinning the variant to sample D7 in the example. The higher the u-score the more likely it is that the signal is due to a true variant. |

Variants with uniqueness scores of 10 or higher were considered pinnable to a specific patient. Next, the variant was assessed for call quality or confidence. A confidence score of up to 100% was calculated using the following approach:

1. Confidence scoring
   1. Using the mean VAF (row batch VAF added to column batch VAF divided by two), proximity to the theoretical allele contribution, gave the 90% of the confidence score. Variants with mean VAFs that exceeded the theoretical allele contribution were designated at 100% of the expected allele contribution. **Example:** A variant with 4% VAF has 80% of the expected allele contribution, which, weighted by 90%, gives 72% confidence. For each variant, this value was added to the value below.
   2. Uniqueness score (excess uniqueness score above the threshold), was weighted with 10% of the confidence. **Example:** A variant with a uniqueness score of 19 is 9 points out of a possible 15, or 60%, in excess of the threshold, which weighted by 10% gives 6% confidence. For each variant, this value was added to the value above.
2. Confidence grouping (added to all variants with a uniqueness score above the threshold)
   1. **No confidence:** variants with confidence score below 24.99%.
   2. **Low confidence:** variants with confidence score of 25.00% to 49.99%.
   3. **Medium confidence:** variants with confidence score of 50.00% to 74.99%.
   4. **High confidence:** variants with confidence score of 75.00% to 100%.
   5. Example: Using the example confidence scores from above, the variant’s confidence score comes to 78% (72% plus 6%); a high confidence variant.

The parameters used in the methods above were a product of empirical testing with an aim of leaving a margin for error/data variation. The analysis of the explorative cohort suggested that the iterative cut-off method outlined above was the most accurate. Following the results of the explorative cohort, we ran a second identical experiment in a validation cohort, containing 100 new patients. The analysis was blinded to the WGS data, and investigators were thus unaware of which patient carried genotypes of interest. The iterative method was used for variant calling in a manner identical to the one informed by the explorative cohort.

After data from the validation cohort were run using the specified cut-offs (for filtering) and quality proportions (for confidence scoring) that were determined by results from the explorative cohort, validation results were cataloged, and shared with an internal unblinded researcher. After this step, the validation cohort matrix data was annotated with WGS data, for benchmarking analysis (Fig. S5).

| 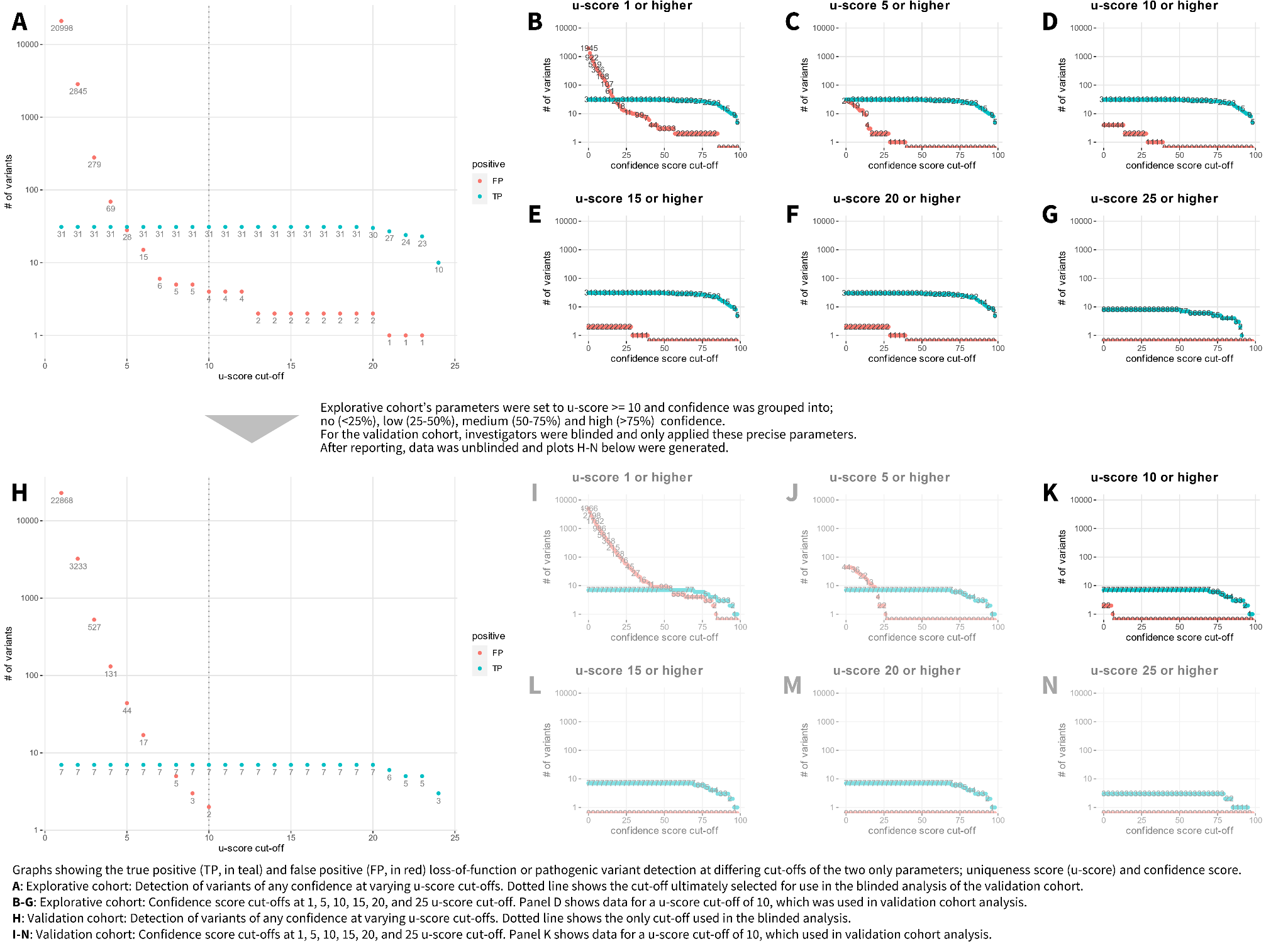 |
| --- |
| **Fig. S5:** Graphs showing the true positive (TP, in teal) and false positive (FP, in red) loss-of-function or pathogenic variant detection at differing cut-offs of the two only parameters; uniqueness score (u-score) and confidence score.  **A**: Explorative cohort: Detection of variants of any confidence at varying u-score cut-offs. The dotted line shows the cut-off ultimately selected for use in the blinded analysis of the validation cohort. **B-G**: Explorative cohort: Confidence score cut-offs at 1, 5, 10, 15, 20, and 25 u-score cut-off. Panel D shows data for a u-score cut-off of 10, which was used in validation cohort analysis. **H**: Validation cohort: Detection of variants of any confidence at varying u-score cut-offs. The dotted line shows the only cut-off used in the blinded analysis.  **I-N**: Validation cohort: Confidence score cut-offs at 1, 5, 10, 15, 20, and 25 u-score cut-off. Panel K shows data for a u-score cut-off of 10, which was used in validation cohort analysis. |

### Statistics

R package EpiR (version 2.0.40) was used for computing true and apparent prevalence, sensitivity, specificity, positive and negative predictive values and positive and negative likelihood ratios as well as exact binomial confidence limits based on count data in a 2 by 2 table format.

## Other supplemental data

### Filtering results

| 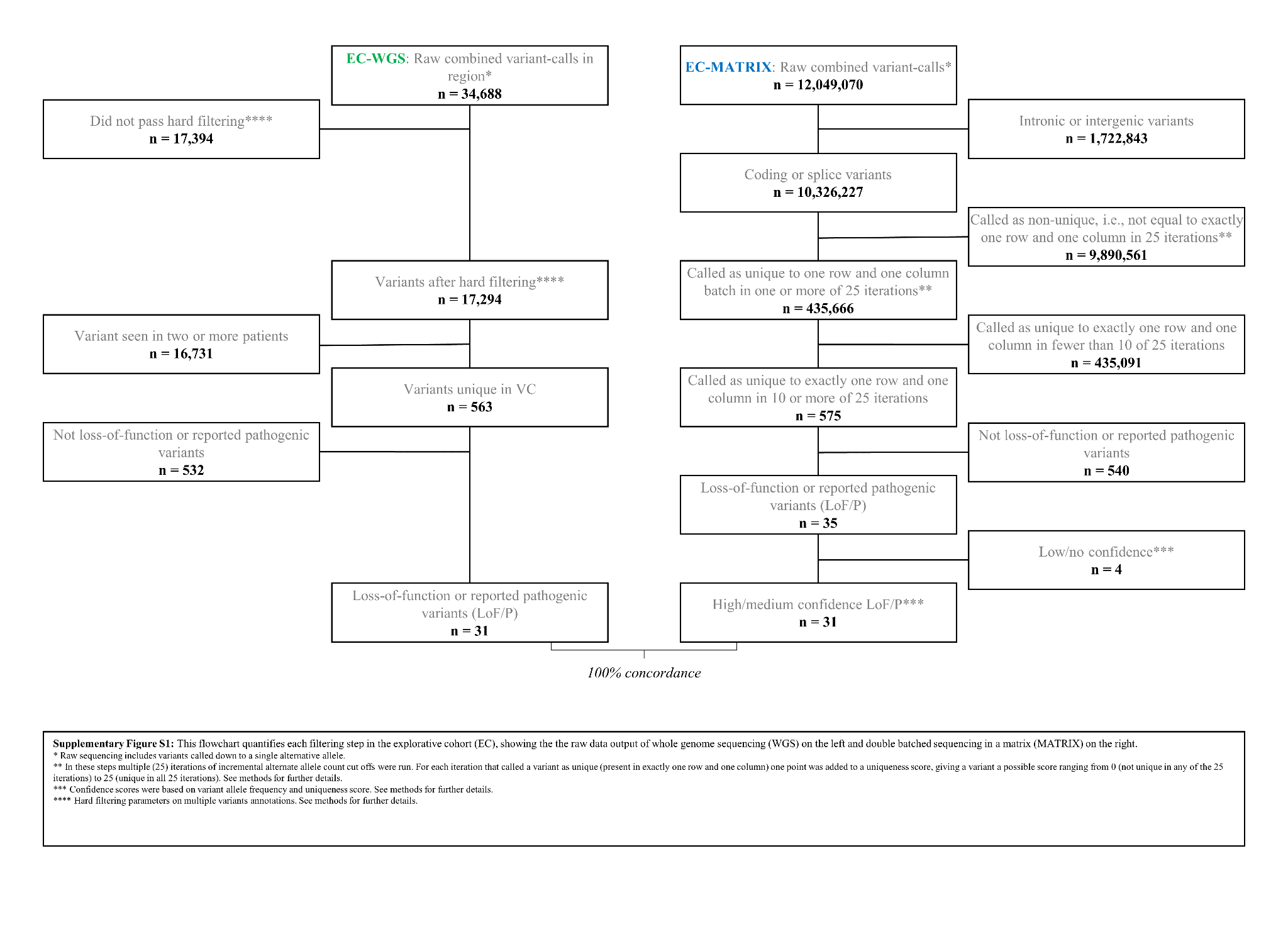 |
| --- |
| **Fig. S6:** This flowchart quantifies each filtering step in the explorative cohort (EC), showing the raw data output of whole genome sequencing (WGS) on the left and double batched sequencing in a matrix (MATRIX) on the right.  * Raw sequencing includes variants called down to a single alternative allele.  ** In these steps multiple (25) iterations of incremental alternate allele count cut-offs were run. For each iteration that called a variant as unique (present in exactly one row and one column) one point was added to a uniqueness score, giving a variant a possible score ranging from 0 (not unique in any of the 25 iterations) to 25 (unique in all 25 iterations). See Methods for further details.  *** Confidence scores were based on variant allele frequency and uniqueness score. See Methods for further details.  **** Hard filtering parameters on multiple variants annotations. See Methods for further details. |

| 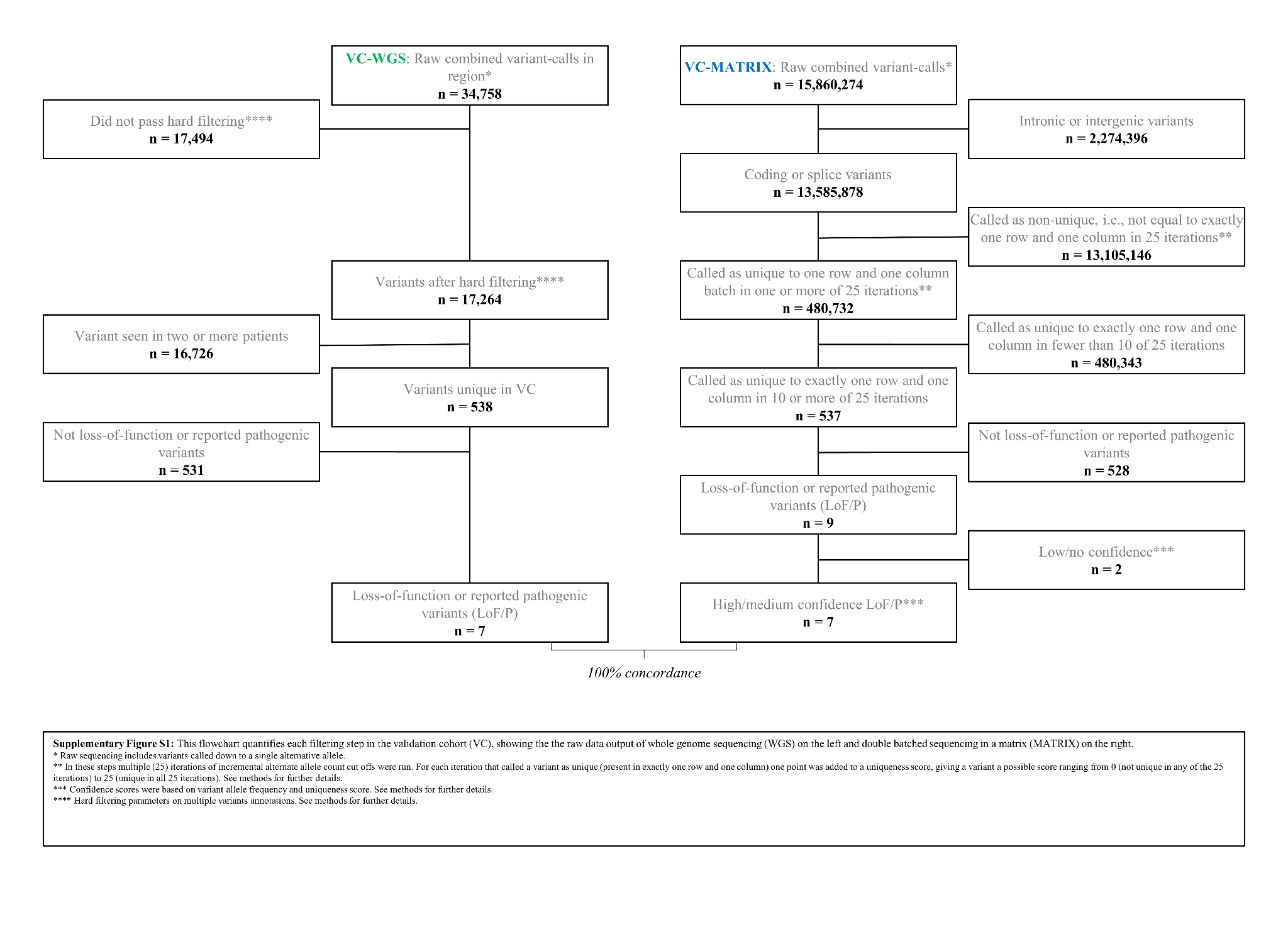 |
| --- |
| **Fig. S7:** This flowchart quantifies each filtering step in the validation cohort (VC), showing the raw data output of whole genome sequencing (WGS) on the left and double batched sequencing in a matrix (MATRIX) on the right.  * Raw sequencing includes variants called down to a single alternative allele.  ** In these steps multiple (25) iterations of incremental alternate allele count cut offs were run. For each iteration that called a variant as unique (present in exactly one row and one column) one point was added to a uniqueness score, giving a variant a possible score ranging from 0 (not unique in any of the 25 iterations) to 25 (unique in all 25 iterations). See methods for further details.  *** Confidence scores were based on variant allele frequency and uniqueness score. See methods for further details.  **** Hard filtering parameters on multiple variants annotations. See methods for further details. |

### All unique variants regardless of clinical relevance

While the primary outcome of this study was to test the ability of DoBSeq in identifying clinically relevant rare variants, we also tested its performance for all variants unique in either of the two cohorts. Here we applied the exact filtering and variant calling/confidence scoring outlined above.

In the explorative cohort, WGS data called a total of 812 variants as unique (seen only once in the cohort). Of these, 560 variants passed QC (described above, three *WT1* variants were excluded due to low coverage on panel) and were considered high-confidence (see Fig. S6). DoBSeq correctly identified 553 variants (true positives, 19 variants had been flagged as low confidence based on WGS data), identified 6 variants not found by WGS (false positives) and failed to identify 26 variants considered high confidence on WGS (false negatives). Of the 26 false negative variants, 17 (65%) were non-coding (i.e. splice or untranslated regions), perhaps poorly captured by the targeted panel sequencing used for DoBSeq. Among the remaining nine false negative variants, two were in *PMS2*, in which NGS calls are known to be hampered by pseudogene variants[^2^](https://www.zotero.org/google-docs/?5n995M), while seven were either missense (4) or silent (3). It is possible that panel-based sequencing may limit pseudogenic capture, such as of the *PMS2* pseudogene, however, we did not specifically test for this, just as we did not attempt Long Range PCR validation of the two *PMS2* variants found using WGS data.

In the validation cohort, WGS data called a total of 793 as unique (seen only once in the cohort). Of these, 537 variants passed QC (described above, three *WT1* variants were excluded due to low coverage on panel) and were considered high-confidence (see Fig. S7). DoBSeq correctly identified 505 variants (true positives, five variants had been flagged as low confidence based on WGS data), identified five variants not found by WGS (false positives) and failed to identify 37 variants considered high confidence on WGS (false negatives). Of the 37 false negative variants, 15 (41%) were non-coding (i.e. splice or untranslated regions), perhaps poorly captured by the targeted panel sequencing used for DoBSeq. Among the remaining 22 false negative variants, all were either missense (13), silent (8), in-frame deletion (1).

Upon manual curation of the 63 false negative variants, some likely causes of falseness emerged. As a whole, 30 false negative variants were undetected in the row batch exclusively (4), the column batch exclusively (2), or both (24). As remarked, a total of 32 false negative variants were designated as non-coding. In 16 cases, these variants were detected in both row and column batches, yet, erratically low coverage, high variant allele frequency, high strand bias, low uniqueness score, or a combination hereof led to the detected variants’ exclusion during filtration. The two *PMS2* were

Of specific interest were the remaining 29 variants, mentioned above; one in-frame deletion and 28 substitution variants, either missense (17) or silent (11). These fell into seven primary groups; high strand bias (7), no detection in either row or column (6), low coverage (4), low uniqueness score (4), no detection in either row or column likely due to low DNA contribution (4, only in sample #J9 in the validation cohort), no detection in row exclusively (3), no detection in column exclusively (1). For full details on false negative variants see Additional file 2: Table S5. From this it appears evident that at least a portion of the false negative variants was a consequence of hard filtering parameters. These parameters were optimized for the primary outcome; LoF/P variant detection. We did not attempt altering the parameters, using only the parameters defined following the explorative cohort analysis (full.

Taken together, DoBSeq correctly identified high confidence unique variants as determined by WGS data at a rate of 94% (1034/1097) and 97% (1034/1066) when discounting non-coding and pseudogenic false negatives. True negatives are not meaningful to consider as any one loci identified by both WGS and DoBSeq as wildtype or reference may be considered true negatives and these count in the hundreds of thousands. As a whole, our findings suggest that DoBSeq’s performance is non-inferior to variant-calling concordance of conventional individual sequencing cross-platform comparisons[^3^](https://www.zotero.org/google-docs/?Pb3RFO).

| **Cohort** | **WGS** | **TP** | **FP** | **FN** | **TN** | **SENS (95%CI)** | **PPV** |
| --- | --- | --- | --- | --- | --- | --- | --- |
| Explorative | 560 | 534 | 6 | 26 | N/A* | 95.36% (93.27% -96.95%) | 98.89% (98.87% - 98.91%) |
| Validation | 537 | 500 | 5 | 37 | N/A* | 93.55% (91.22% - 95.42%) | 99.08% (99.06% - 99.10%) |
| Combined | 1097 | 1034 | 11 | 63 | N/A* | 94.26% (92.71% - 95.56%) | 98.95% (98.93% - 98.96%) |

**Table S2:** Test statistics showing, WGS, whole genome sequencing variants identified as unique to the cohort and high-confidence, TP, number of true positive variants [reliably found by DoBSeq], FP, number of false positive variant [found by DoBSeq, but not by WGS], FN, number of false negative variants [found by WGS, but not by DoBSeq], TN, true negative variants include all other genetic loci (>400,000) and *) are not considered here, SENS, sensitivity, CI, confidence interval, PPV, positive predictive value. Each metric is shown for the explorative cohort and the validation cohort, as well as those two combined.

### DNA extraction yields

As described in the Methods section, DNA was extracted from two 3.2 mm discs stamped out of the participants original neonatal dried blood spot collected at birth. In the explorative and validation cohorts, the DNA extraction yielded a mean 185.9ng [range; 70ng to 328ng] and 165.0ng [range; 5.2ng to 593ng] of genomic DNA, respectively. On average, DNA yields exceeded the amount required (two times 10ng) to run the experiment by more than 700%. However, extractions for two samples in the validation cohort had inexplicably low yields, which resulted in a DNA contribution of 2ng and 6ng, rather than the desired 10ng. This minor, known limitation did not meaningfully impact the results, and hence re-extraction of DNA was not pursued. The generally very high yields meant that there was ample DNA to run additional larger batches and any required confirmatory tests, although in a clinical implementation, confirmation would presumably be required in a new, independent blood sample. Full results on DNA yields/concentrations along with volumes used for each samples contribution to a batch are included in Additional file: Table S6 .

### Theoretical cost savings

When DNA sequencing is performed on an individual level the cost of sequencing a population increases linearly with the number of individuals in that population. Effectively the price per sample (PPS) remains constant and may be expressed by the following:

[
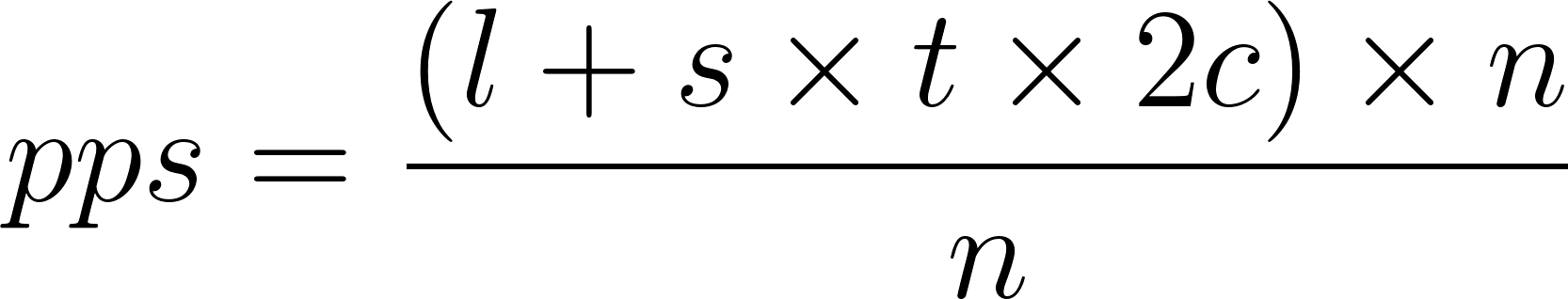
](https://latex-staging.easygenerator.com/eqneditor/editor.php?latex=pps%3D%5Cfrac%7B(l%20%2B%20s%20%5Ctimes%20t%20%5Ctimes%202c)%20%5Ctimes%20n%7D%7Bn%7D#0)

[
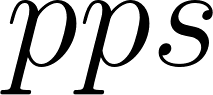
](https://latex-staging.easygenerator.com/eqneditor/editor.php?latex=pps#0) = price per sample

[
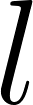
](https://latex-staging.easygenerator.com/eqneditor/editor.php?latex=l#0) = cost of library preparation in USD

[
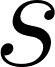
](https://latex-staging.easygenerator.com/eqneditor/editor.php?latex=s#0) = cost of sequencing per megabase in USD

[
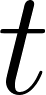
](https://latex-staging.easygenerator.com/eqneditor/editor.php?latex=t#0) = size of targeted genomic regions in megabases

[
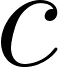
](https://latex-staging.easygenerator.com/eqneditor/editor.php?latex=c#0) = desired coverage per allele

[
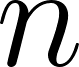
](https://latex-staging.easygenerator.com/eqneditor/editor.php?latex=n#0) = number of individuals in population

Because the one-dimensional batching strategy cannot be used to do direct identification of individuals with a given genotype, it introduces secondary costs that are highly dependent on frequencies of findings. Hence it is not explored further here.

In multi-dimensional batching, the PPS may be expressed by the following:

[
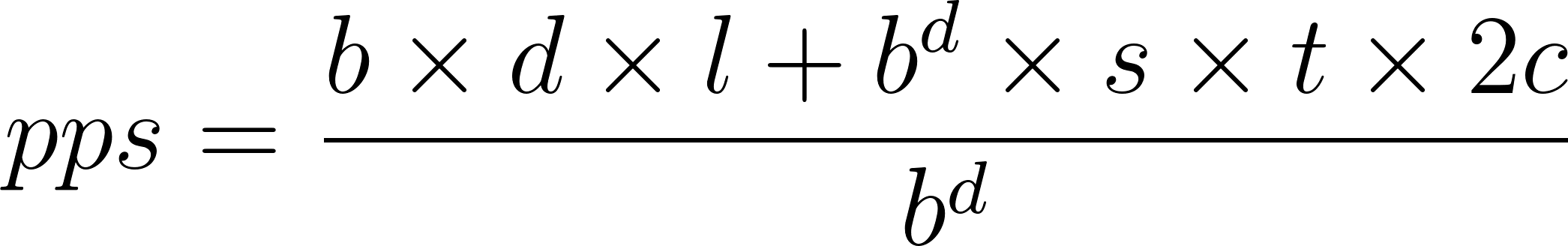
](https://latex-staging.easygenerator.com/eqneditor/editor.php?latex=pps%3D%5Cfrac%7Bb%20%5Ctimes%20d%20%5Ctimes%20l%20%2B%20b%5Ed%20%5Ctimes%20s%20%5Ctimes%20t%20%5Ctimes%202c%7D%7Bb%5Ed%7D#0)

[
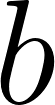
](https://latex-staging.easygenerator.com/eqneditor/editor.php?latex=b#0) = number of batches per dimension

[
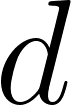
](https://latex-staging.easygenerator.com/eqneditor/editor.php?latex=d#0) = number of dimensions

Assuming that the desired coverage per allele (pure sequencing costs) is constant regardless of approach, the cost savings may be expressed simply as the fraction of library preparations needed:

[
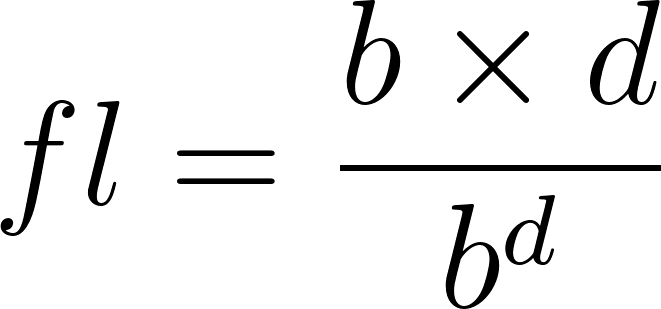
](https://latex-staging.easygenerator.com/eqneditor/editor.php?latex=fl%3D%5Cfrac%7Bb%20%5Ctimes%20d%7D%7Bb%5Ed%7D#0)

[
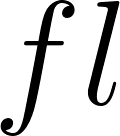
](https://latex-staging.easygenerator.com/eqneditor/editor.php?latex=fl#0) = fraction of library preparations needed compared to individual sequencing

For matrices designed in two dimensions the cost savings would amount to the following (Fig. S8):

[
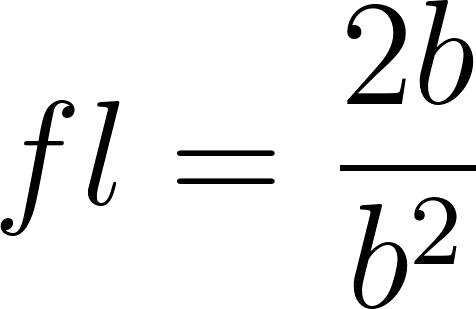
](https://latex-staging.easygenerator.com/eqneditor/editor.php?latex=fl%3D%5Cfrac%7B2b%7D%7Bb%5E2%7D#0)

| 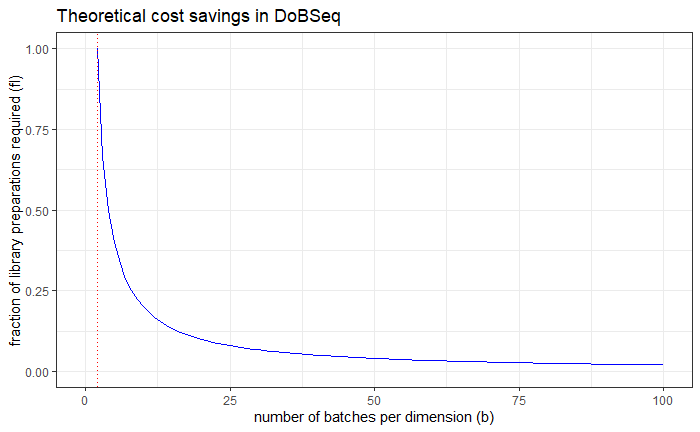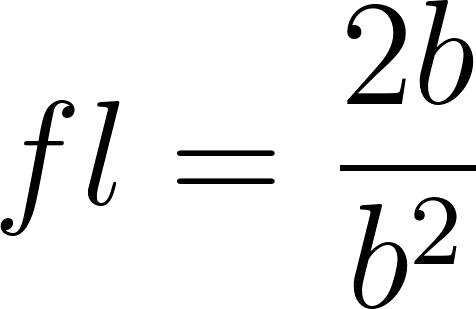 |
| --- |
| **Fig. S8:** Graph illustrating the decreasing fraction of library preparations required ($fl$) as the number of batches per dimension ($b$) increases. At two batches per dimension, a total of four library preparations are required to sequence all samples twice. four is the same number of library preparations needed to sequence individually, hence the $fl$ is 1.00 (red dotted line). Starting with a matrix of 3x3 the $fl$ decreases by continuously smaller fractions for each time $b$ is expanded. Thus, the library preparations required are reduced to 50% in a 4x4 matrix, 25% in an 8x8 matrix, 12.5% in a 16x16 matrix, 6.25% in a 32x32 matrix, etc. Consequently, the $fl$ variable approaches an asymptote of 0 as $b$increases towards infinity. |

### Calculated cost savings

In order to calculate costs, we used pricing examples from our setups (list prices were collected from <https://research.ncsu.edu/gsl/pricing/> ; accessed October 31st 2022). Prices of kits, sequencing and all other direct and in-direct costs tend to vary greatly between and even within countries and institutions. Hence our calculations and estimates serve merely to illustrate the main costs and savings within our setup.

As explained in Methods, we used an extraction kit targeting a genomic region of 403 kilobases. Using list prices of the NovaSeq 6000 platform employing an S4 flow-cell and Reagent Kit v1.5 at 300 cycles, the expected cost per gigabase is 5.90 USD (list prices October 31st 2022) (Main manuscript: Table 2). This equals a cost per 1X of coverage for the 403 kilobase region of 0.002 USD. At the desired coverage per allele of 100X, the estimated cost of sequencing per individual equals 0.4 USD. This underscores the low cost associated with the DNA sequencing itself. Running smaller, less cost-effective flow cells may result in sequencing costs as high as 3.8 USD per sample, however, large flow cells are required when DoBSeq is run at scale (Main manuscript: Table 2).

Conversely, the off-the-shelf cost of the sequencing library preparation kit (incl. enrichment and indexing) was 102.4 USD. As shown above, DoBSeq reduces the number of library preps needed to sequence a population (Fig. S8). We tested this with two full matrices at 10 times 10 batches. We also tested the sensitivity at increased batch sizes (stand-alone batches) at 24, 48, 72, and 96 individuals per batch, without increasing the total coverage per batch, yielding a linearly decreasing coverage per sample.

Our robustly tested setups of 10 times 10 matrices yield a PPS of approx. 20 USD. The empirical data indicates that the data generated even at a PPS as low as 3 USD is likely to have fully satisfactory performance, however, this ultimately requires empirical testing using DoBSeq in a larger cohort.

### Analysis of scaled batches

The four stand-alone batches containing 24, 48, 72 and 96 EC participants were sequenced to a depth of 83X, 42X, 28X, 21X per sample, respectively. A sensitivity-only analysis was done to test the increased batches’ ability to detect the LoF/P variants known to be present based on WGS data. Due to the high number of false positives in single batch sequencing we also tested detection of all cohort-unique variants known *not* to be in the batch. The VAFs among LoF/P variants known to be in the batches matched the expected VAF, while the small fraction of calls in variants that were known not to be in the batch were substantially lower (Table S2).

As the DoBSeq method is scaled, i.e., applied on matrices with increasing sample sizes, the likelihood that a matrix will contain two or more carriers of identical variants also increases. When considered as a standalone analysis (i.e., without additional individuals sequencing tests), DoBSeq provides a conclusive result regarding the carrier statuses for all individuals only when a variant is not present or is uniquely present in one individual. The likelihood of detecting two or more individuals in a matrix is highly dependent on the minor allele frequency in the tested population (Table S3).

| **Size of DoBSeq setup** | | | **Minor allele frequency** | | | | |
| --- | --- | --- | --- | --- | --- | --- | --- |
| **Batch size** | **Sample size** | **Allele number** | **0.0001%** | **0.001%** | **0.01%** | **0.1%** | **1%** |
| 10 | 100 | 200 | 100% | 100% | 100% | 99.5% | 73.6% |
| 24 | 576 | 1,152 | 100% | 100% | 99.8% | 88.6% | 2.09% |
| 48 | 2,304 | 4,608 | 100% | 100% | 97.7% | 33.0% | 0.00% |
| 72 | 5,184 | 10,368 | 100% | 99.9% | 90.4% | 3.46% | 0.00% |
| 96 | 9,216 | 18,432 | 100% | 99.6% | 76.5% | 0.10% | 0.00% |

**Table S3:** Theoretical proportion of matrices where DoBSeq should provide conclusive results (either a pin-pointed unique variant or no variant) given the matrix size and the minor allele frequency of the variant in the test population.


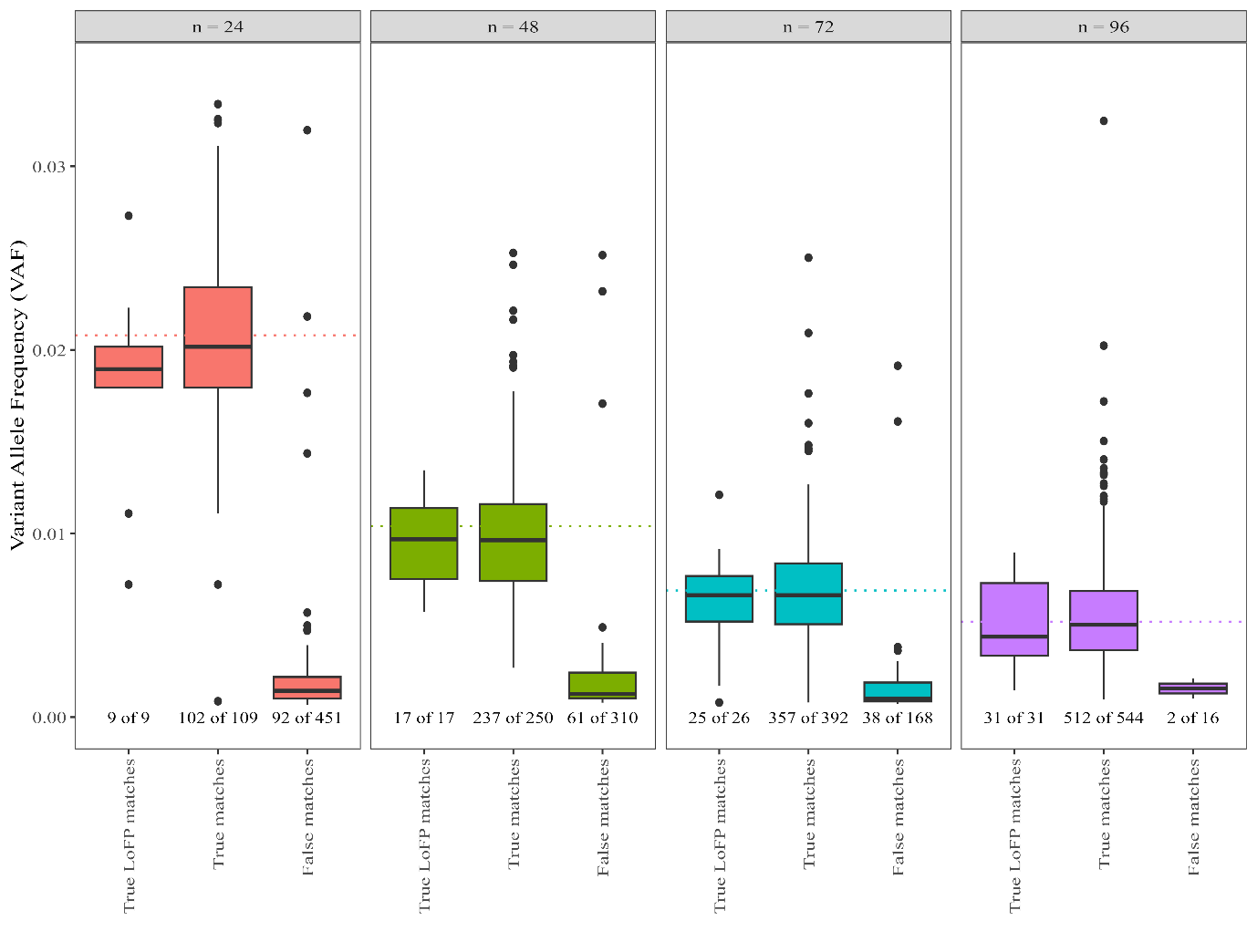


**Fig. S9:** Box plot of the variant allele frequency (VAF) for three groups: True loss-of-function / known pathogenic (LoFP) matches, True matches, and False matches. Using whole-genome sequencing (WGS) data as a benchmark, all variants are unique to one individual in the cohort. The plot is faceted by batch size indicated by the label "n = x" where x is the number of samples in the batch. The number of variant in each group is annotated at the bottom of each panel, with the format "y of z". For true matches, y is the number of variants seen in the stand-alone batch and z is the number of variants seen in the WGS data. For false matches, y is the number of variants seen in the stand-alone batch out of z, which are all the unique cohort variants *not* included in the batch. The dotted line represents the theoretical VAF contribution of one allele for each batch size.

|  |
| --- |

| 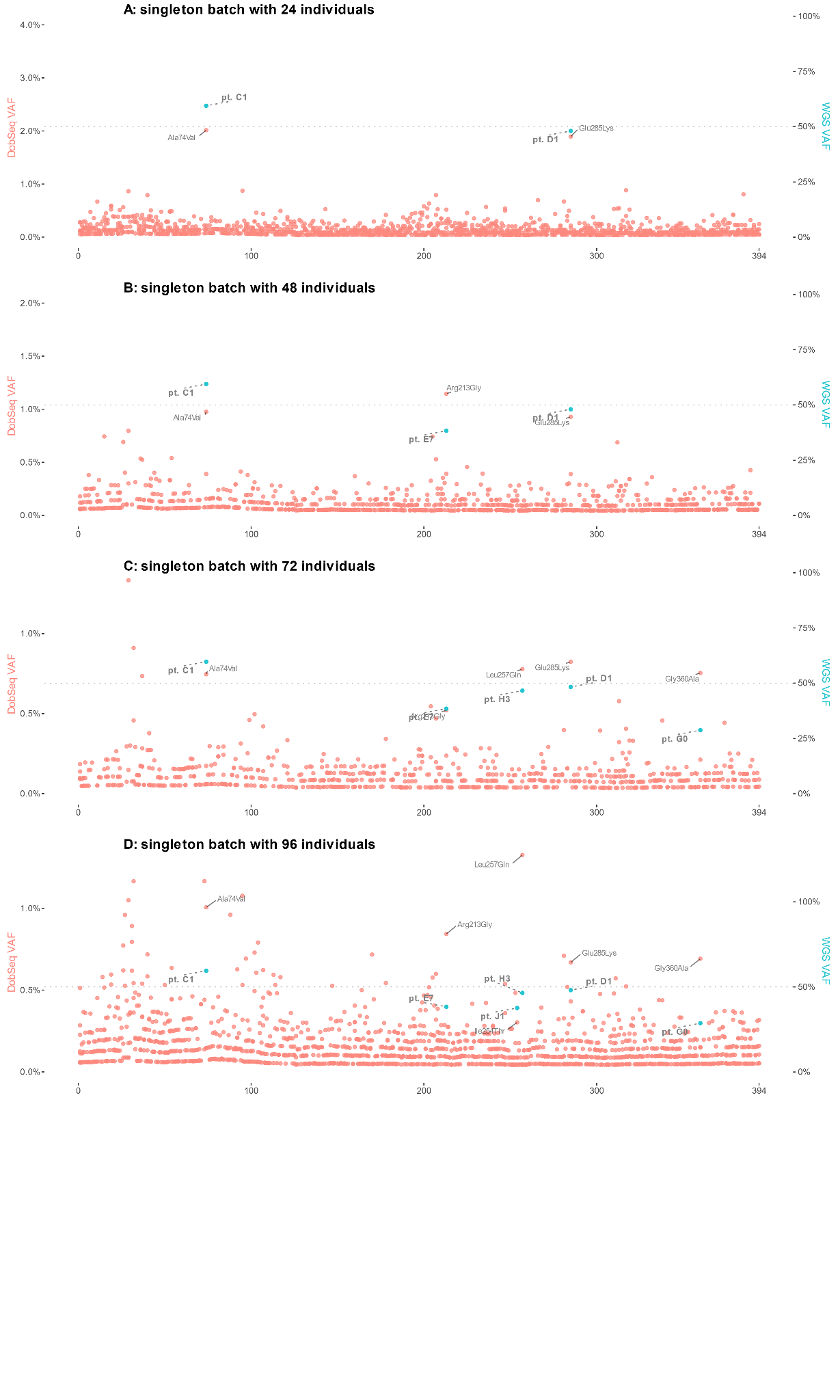 |
| --- |
| **Fig. S10:** Reidentifying *TP53* variants in stand-alone batches with 24, 48, 72 and 96 individuals, respectively. Total coverage was kept constant leading to a decreasing coverage per allele.  **A:** Illustrates all non-synonymous coding variants in the *TP53* gene called without any filtering, i.e. includes all low-coverage and low-confidence calls, showing DoBSeq variants found in a single batch of 24 individuals from the explorative cohort (EC) in red and whole genome sequencing (WGS) variants found in the same individuals in teal. The x axis shows the codon positions of the canonical *TP53* protein product. The y axis shows variant allele frequency (VAF) for DoBSeq on the left and WGS on the right. The dotted line indicates the theoretical VAF for true heterozygous variants [2.08%; 50%]. A common polymorphism (p.Pro72Arg) was filtered out for clarity.  **B:** Same as above for the stand-alone batch containing 48 individuals. The theoretical VAF for true heterozygous variants in the batch was 1.04%.  **C:** Same as above for the stand-alone batch containing 72 individuals. The theoretical VAF for true heterozygous variants in the batch was 0.69%.  **D:** Same as above for the stand-alone batch containing 96 individuals. The theoretical VAF for true heterozygous variants in the batch was 0.52%.  *Interpretation:* A steady level of sequencing noise runs along the low end of the y axis with individual calls in the stand-alone batches rising up towards or slightly above the expected VAF. This allows for detection, but not pin-pointing, of variants. As the number of samples per batch increases and coverage per allele decreases, true and false variants become increasingly difficult to separate. In the larger batches true variants tend to exceed the expected VAF, likely due to sequencing errors compounding to the true allele contribution. |

|  |
| --- |

|  |
| --- |

### Cost-effectiveness: Adult CPSs

| \| 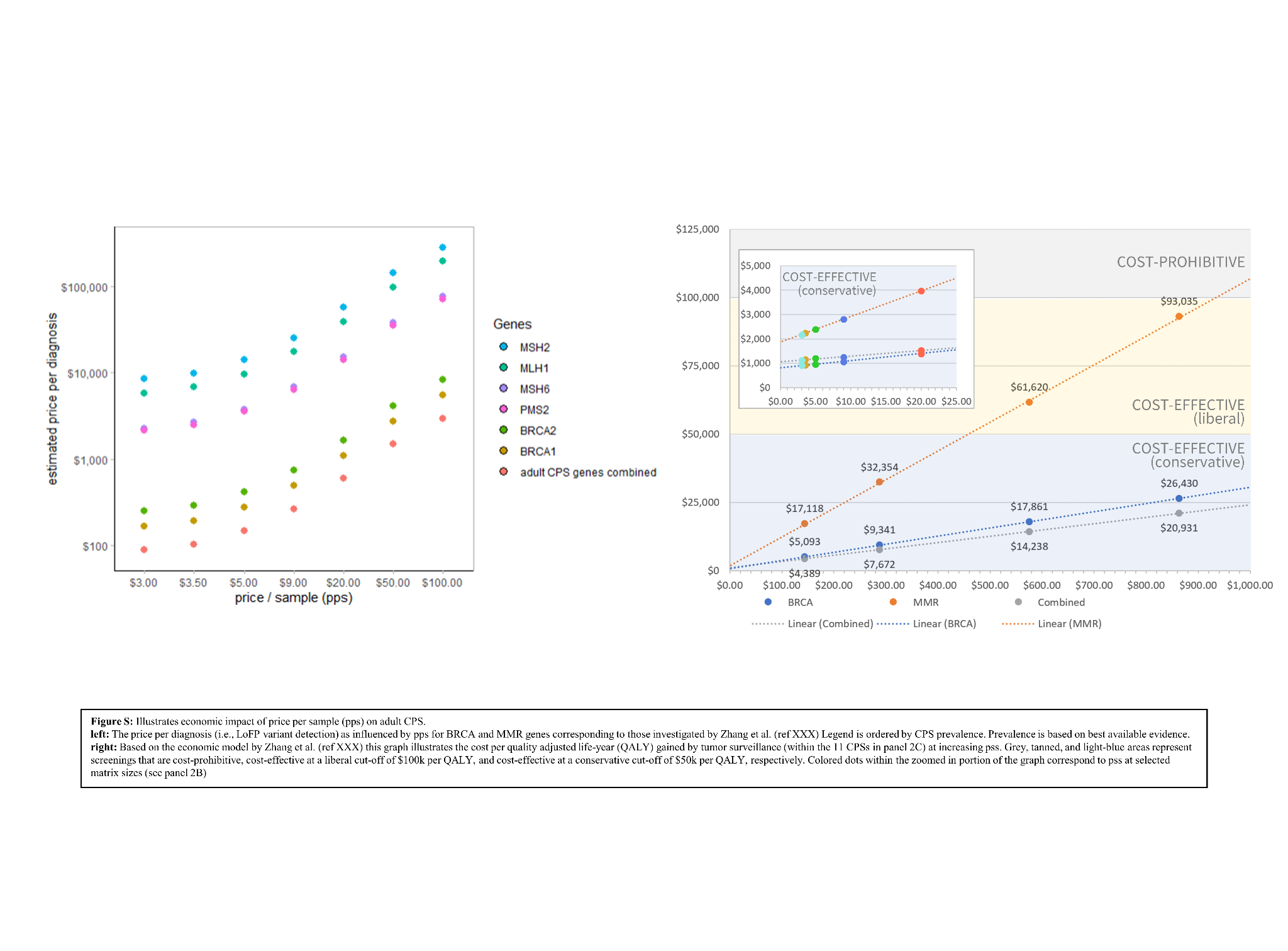 \| \| --- \| \| **Fig. S11:** Illustrates economic impact of price per sample (PPS) on adult cancer predisposition syndrome (CPS) genes. The price per diagnosis (i.e., LoF/P variant detection) as influenced by PPS for *BRCA* and mismatch repair (MMR; *MSH2*, *MLH1*, *MSH6*, and *PMS*) genes corresponding to those investigated by Zhang et al[^4^](https://www.zotero.org/google-docs/?vbl3sp). Legend is ordered by CPS prevalence. Prevalence is based on best available evidence. \| |
| --- | --- | --- |
| \| 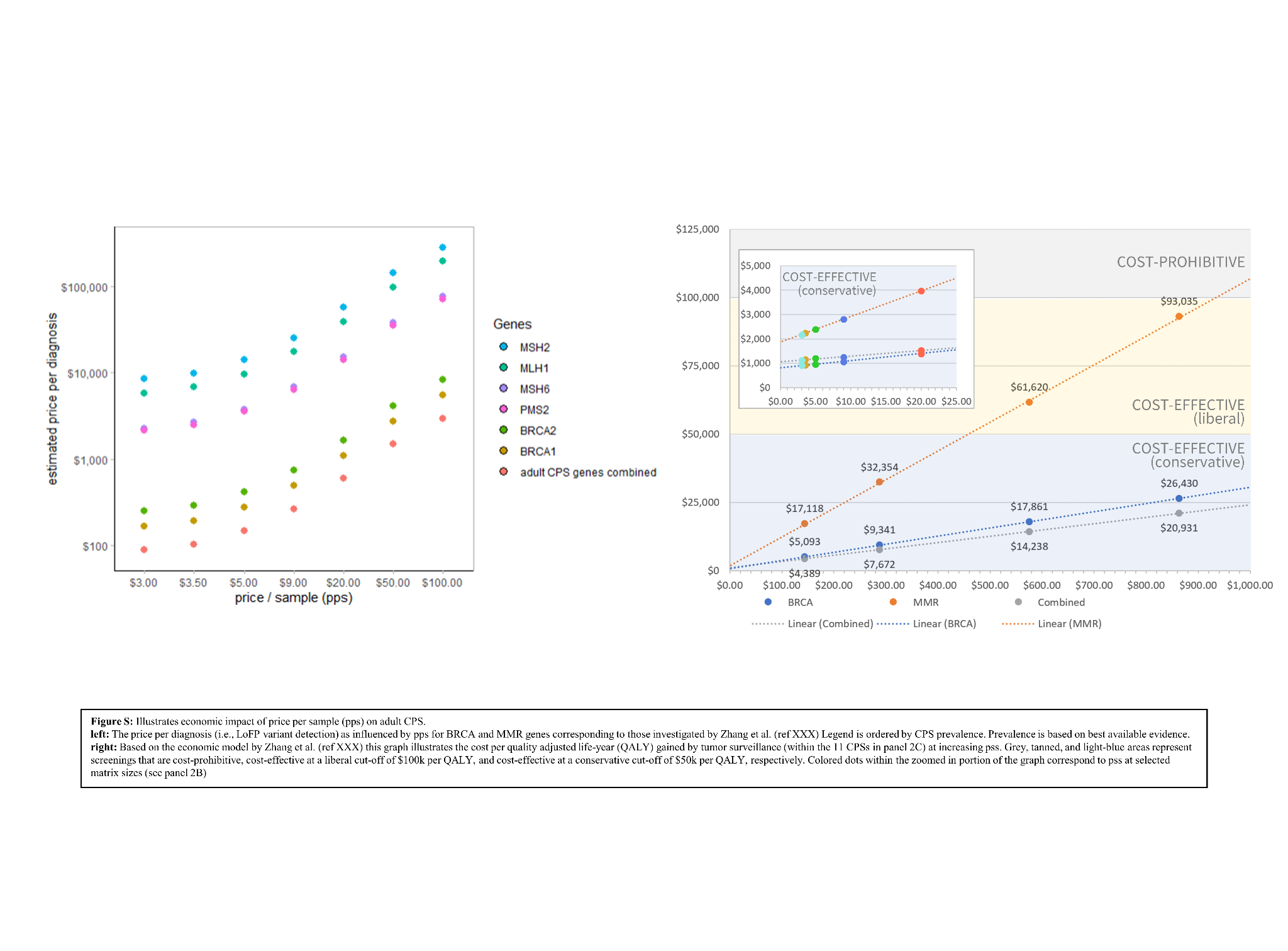 \| \| --- \| \| **Fig. S12:** Illustrates economic impact of price per sample (PPS) on adult cancer predisposition syndromes (CPS). Based on the economic model by Zhang et al.[^4^](https://www.zotero.org/google-docs/?cRYBdv) this graph illustrates the cost per quality adjusted life-year (QALY) gained by tumor surveillance (within the *BRCA* and mismatch repair (MMR; *MSH2*, *MLH1*, *MSH6*, and *PMS*) related CPSs) at increasing PPS. Gray, tanned, and light-blue areas represent screenings that are cost-prohibitive, cost-effective at a liberal cut-off, corresponding to $100k per QALY, and cost-effective at a conservative cut-off of $50k per QALY, respectively. Colored dots within the zoomed in portion of the graph correspond to PPS at selected matrix sizes (colors correspond to Fig. 3B). \| |

| 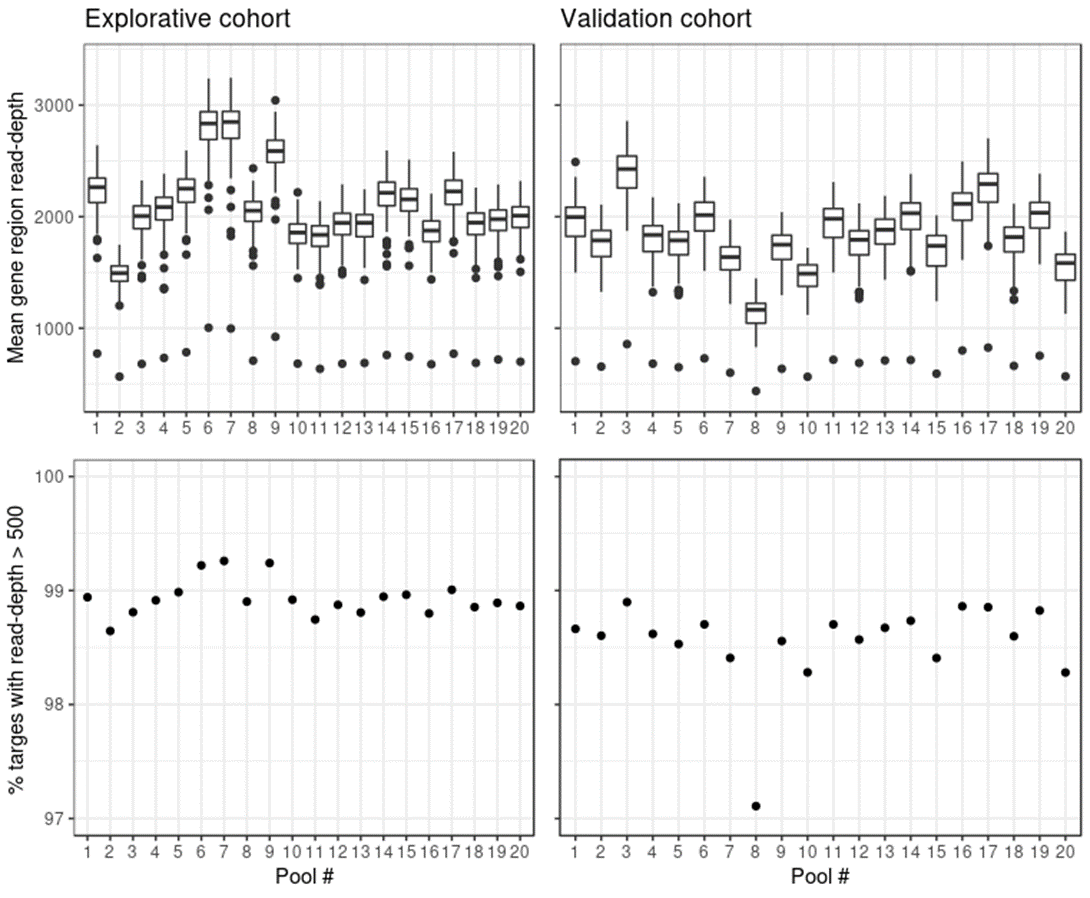 |
| --- |
| **Fig. S13:** Illustrates coverage metrics for each pool in the explorative and validation cohorts.  **Upper panels:** Mean coverage for each of the 113 genes covered by the panel shown for each pool both the explorative and validation cohort.  **Lower panels:** Percentage of the entire target region covered to at least 500X shown for both the explorative and validation cohort. |

##

## References

[1. Byrjalsen, A., Hansen, T.V.O., Stoltze, U.K., Mehrjouy, M.M., Barnkob, N.M., Hjalgrim, L.L., Mathiasen, R., Lautrup, C.K., Gregersen, P.A., Hasle, H., et al. (2020). Nationwide germline whole genome sequencing of 198 consecutive pediatric cancer patients reveals a high incidence of cancer prone syndromes. PLoS Genet. *16*. 10.1371/journal.pgen.1009231.](https://www.zotero.org/google-docs/?MKKIgg)

[2. Stoltze, U.K., Foss-Skiftesvik, J., van Overeem Hansen, T., Byrjalsen, A., Sehested, A., Scheie, D., Mikkelsen, T.S., Rasmussen, S., Bak, M., Okkels, H., et al. (2022). Genetic predisposition & evolutionary traces of pediatric cancer risk: A prospective 5-year population-based genome sequencing study of children with CNS tumors. Neuro-Oncol., noac187. 10.1093/neuonc/noac187.](https://www.zotero.org/google-docs/?MKKIgg)

[3. O’Rawe, J., Jiang, T., Sun, G., Wu, Y., Wang, W., Hu, J., Bodily, P., Tian, L., Hakonarson, H., Johnson, W.E., et al. (2013). Low concordance of multiple variant-calling pipelines: practical implications for exome and genome sequencing. Genome Med. *5*, 28. 10.1186/gm432.](https://www.zotero.org/google-docs/?MKKIgg)

[4. Zhang, L., Bao, Y., Riaz, M., Tiller, J., Liew, D., Zhuang, X., Amor, D.J., Huq, A., Petelin, L., Nelson, M., et al. (2019). Population genomic screening of all young adults in a health-care system: a cost-effectiveness analysis. Genet. Med. *21*, 1958–1968. 10.1038/s41436-019-0457-6.](https://www.zotero.org/google-docs/?MKKIgg)
